# StarD13 negatively regulates invadopodia formation and invasion in high-grade serous (HGS) ovarian adenocarcinoma cells by inhibiting Cdc42

**DOI:** 10.1016/j.ejcb.2021.151197

**Published:** 2021-12-21

**Authors:** Sandra Abdellatef, Isabelle Fakhoury, Maria Al Haddad, Leila Jaafar, Hiba Maalouf, Samer Hanna, Bassem Khalil, Zeinab El Masri, Louis Hodgson, Mirvat El-Sibai

**Affiliations:** a Department of Natural Sciences, School of Arts and Sciences, Lebanese American University, Beirut, Lebanon; b Department of Pediatrics Hematology/Oncology division, Weill Cornell Medicine, Joan & Sanford I. Weill Medical College of Cornell University, Ithaca, NY, USA; c Department of Medicine, Icahn School of Medicine at Mount Sinai, Department of Biological Sciences, Fordham University, Bronx, NY, USA; d Department of Biochemistry and Molecular Biology, University Park, Pennsylvania State University, State College, PA, USA; e Department of Anatomy and Structural Biology, Albert Einstein College of Medicine of Yeshiva University, Bronx, NY, USA; f Gruss-Lipper Biophotonics Center, Albert Einstein College of Medicine of Yeshiva University, Bronx, NY, USA

**Keywords:** StarD13, Ovarian cancer, Cell invasion, Invadopodia, Cdc42

## Abstract

Metastasis remains the main challenge to overcome for treating ovarian cancers. In this study, we investigate the potential role of the Cdc42 GAP StarD13 in the modulation of cell motility, invasion in ovarian cancer cells. StarD13 depletion does not affect the 2D motility of ovarian cancer cells. More importantly, StarD13 inhibits matrix degradation, invadopodia formation and cell invasion through the inhibition of Cdc42. StarD13 does not localize to mature TKS4-labeled invadopodia that possess matrix degradation ability, while a Cdc42 FRET biosensor, detects Cdc42 activation in these invadopodia. In fact, StarD13 localization and Cdc42 activation appear mutually exclusive in invadopodial structures. Finally, for the first time we uncover a potential role of Cdc42 in the direct recruitment of TKS4 to invadopodia. This study emphasizes the specific role of StarD13 as a narrow spatial regulator of Cdc42, inhibiting invasion, suggesting the suitability of StarD13 for targeted therapy.

## 1. Introduction

Ovarian cancer is the second most common cancer of the female reproductive system and the primary cause of death of patients with female reproductive system malignancies (Hunn and Rodriguez, 2012). The American Cancer Society estimates that there will be around 21,750 new cases of ovarian cancer and 13,940 deaths from ovarian cancer in the United States in 2020 (Hunn and Rodriguez, 2012). Tumor metastasis from the ovaries to a secondary site remains the main cause of death in patients with ovarian cancer (Yeung et al., 2015). Hence, there is a need to understand the mechanisms regulating ovarian cancer cell motility and invasion as well as identifying the key molecular players involved in these processes for effective treatment of ovarian cancer.

Metastasis is a result of the dysregulation of molecules that regulate actin polymerization and cell migration, leading to the acquired ability of cancer cells to metastasize and invade (Giese et al., 2003; Nakada et al., 2007). Initially, the cell protrudes towards the direction of the chemoattractant through de novo actin polymerization (Bailly et al., 1998). The cell adheres to the substratum, thereby committing to movement (Gupton and Waterman-Storer, 2006), moves itself forward through actomyosin contractility and finally detaches its tail in order to propel itself forward (Condeelis et al., 2001). Following initial migration, cancer cells form 3D invasive structures called invadopodia, which possess matrix metalloproteinase activity. This enables cancer cells to degrade the extracellular matrix and to invade the surrounding tissues (Artym et al., 2006; Gimona and Buccione, 2006; Yamaguchi et al., 2005). Invadopodia are F-actin-rich vertical protrusions, that contain regulators of the actin cytoskeleton, such as cortactin, cofilin, Arp2/3 and matrix metalloproteinases (Artym et al., 2006; Gimona and Buccione, 2006; Oser and Condeelis, 2009; Oser et al., 2009; Yamaguchi et al., 2005), as well as members of the Rho family of small guanosine triphosphatases (GTPases) (Sakurai-Yageta et al., 2008).

Rho GTPases are small monomeric G proteins that belong to the Ras superfamily (Takai et al., 2001). These are molecular switches that regulate all signal transduction pathways involving the reorganization of the actin cytoskeleton (Ridley and Hall, 1992). Almost all processes and events of tumor cell proliferation, motility and invasion including cellular polarity, cytoskeletal re-organization, and signal transduction pathways are controlled through the interplay between the different Rho-GTPases (El Atat et al., 2019; Hanna and El-Sibai, 2013; Sahai and Marshall, 2002; Tang et al., 2008). Rho GTPases are found in two forms, a GDP-bound inactive and a GTP-bound active form (Bourne et al., 1990). Rho GTPases are regulated by three classes of proteins, Guanine nucleotide exchange factors (GEFs), GTPase-activating proteins (GAPs), and guanine nucleotide dissociation inhibitors (GDIs). GAPs negatively regulate Rho GTPases by stimulating the intrinsic GTPase activity of Rho GTPases and promoting the formation of the inactive GDP-bound form (Moon and Zheng, 2003).

Steroidogenic acute regulatory protein-related lipid transfer domain-containing protein 13 (StarD13) is a GAP for RhoA and Cdc42 with potential tumor suppressor functions (Ching et al., 2003; El-Sitt and El-Sibai, 2013; El-Sitt et al., 2012; Ullmannova and Popescu, 2006). Indeed, several studies have shown that StarD13 is under expressed in many types of tumors, and that the overexpression of StarD3 inhibits cancer cell growth and proliferation (Al Haddad et al., 2020; Ching et al., 2003; El-Sitt et al., 2012; Gao et al., 2018; Yang et al., 2019a). In contrast with its tumor suppressor function however, we have demonstrated that StarD13 positively regulates cell migration of different cancer types including astrocytoma, breast, lung, and colorectal cancer (Al Haddad et al., 2020; Hanna et al., 2014a; Khalil et al., 2014; Nasrallah et al., 2014). However, the expression of StarD13 in ovarian cancer tissues versus normal ovarian tissues as well as the role of StarD13 in ovarian cancer cell proliferation, motility and invasion remains unknown. Therefore, this study aims at comparing the expression of StarD13 in normal and cancerous tissues, analyzing the effects of StarD13 on the migration and invasion of different ovarian cancer cell lines, as well as uncovering the mechanisms and downstream targets mediating the role of StarD13 in ovarian cancer.

The results obtained support the tumor suppressor role of StarD13 and that StarD13 is a GAP for Cdc42 in ovarian cancer cell lines, as was previously shown in other tumor types (Ching et al., 2003). Moreover, we show that StarD13 inhibits cell adhesion, cell protrusions, cell invasion, matrix degradation and the formation of invadopodia of ovarian cancer cells while having no effect on their 2D motility. Furthermore, we dissect, for the first time, the role of StarD13 in the suppression of invadopodia formation, through the suppression of Cdc42 activation. Finally, the data show that RhoA is required for matrix degradation and invasion of ovarian cancer cells independently from StarD13.

## 2. Materials and methods

### 2.1. Antibodies and reagents

Cdc42 and RhoA biosensors were kind gifts from Dr. Louis Hodgson (Albert Einstein College of Medicine). RhoA/Rac1/Cdc42 Activation Assay Combo Kit was purchased from Cell BioLabs (Sand Diego, CA, USA). QCM Gelatin Invadopodia Assay was obtained from Millipore (Massachusetts, USA). Primary antibodies against Cdc42, StarD13, actin, vinculin, Arp2, cortactin (Mouse and Rabbit) and TKS5 antibodies were obtained from Abcam (Cambridge, UK). Primary mouse anti-TKS4 was obtained from Merck Millipore. Primary mouse anti-StarD13 and rabbit anti-Cdc42 were obtained from Santa Cruz Biotech. HRP-conjugated secondary antibodies were obtained from Promega (Wisconsin, USA). Fluorescent secondary antibodies Alexa Fluor 488-green and Alexa Fluor 594-red as well as Rhodamine-phalloidin stain were purchased from Invitrogen (Massachusetts, USA). DAPI stain, and cell proliferation reagent were acquired from Roche Diagnostics (Roche Ltd, Mannheim, Germany). Hiperfect transfection reagent, luciferase and human Flexi Tubes siRNA for luciferase, StarD13, Cdc42 and RhoA were obtained from Qiagen (Hilden, Germany). Lipofectamine LTX was from Waltham (Massachusetts, USA) and crystal violet was from SCP Science (Quebec, Canada).

### 2.2. Cell culture

Metastatic SKOV-3 (Human ovarian adenocarcinoma derived from ovaries and from metastasis in ascites), Caov-3 (Human ovarian adenocarcinoma) and PA-1 (Human ovarian teratocarcinoma derived from metastasis in ascites) cell lines were purchased from ATCC (American Type Culture Collection). All cell lines were culture in DMEM medium supplemented with 10% fetal bovine serum and 100U penicillin/streptomycin at 37 °C and 5% CO_2_.

### 2.3. Expression analysis

To determine the expression of StarD13 in human ovarian tumors, we mined the publicly available Repository Oncoming gene expression microarray database (National Cancer Institute, https://www.oncomine.org/resource/login.html). Data was plotted using the normal versus ovarian cancer data sets and parameters and the threshold was set at p-value of 0.001. The analysis reflects the expression of StarD13 in normal ovarian tissue as well as different groups of ovarian cancers (NT: None tumorigenic, OCCA: ovarian clear cell adenocarcinoma, OEA: ovarian endometrioid adenocarcinoma, OMA: ovarian mucinous adenocarcinoma, OSA: ovarian serous adenocarcinoma). StarD13 gene expression from two different microarray data sets was plotted.

### 2.4. Immunostaining

Ovarian cancer cells were plated on glass coverslips and transfected with siRNA or biosensors as indicated. The media was removed and the cells were washed with PBS1X before fixation with 4% paraformaldehyde for 10 min. Following, the cells were permeabilized for 15 min with 0.5% Triton-X100, and then blocked with 1% BSA blocking solution for 1 h. All cells were incubated with the different primary antibodies overnight at 4 °C and with the appropriate fluorescent secondary alexa fluor antibody for 1 h the day after. Finally, all coverslips were washed and stained with Rhodamine-phalloidin for 30 min before mounting using a mounting solution and sealing the slides. Fluorescent cell images were taken using the 63X objective lens of the fluorescent Zeiss Observer Z1 microscope operated by the Zen software (Oberkochen, Germany).

### 2.5. Immunohistochemistry

Normal ovarian and ovarian adenocarcinoma paraffin-embedded tissues were purchased from OriGene technologies (Maryland, USA). Paraffin was removed by soaking the slides in xylol before rehydrating the tissues with PBS for 20 min at room temperature. Next, 200–300 ml of pre-heated antigen retrieval buffer (1 mM EDTA pH=8) were added to the slides before heating twice for 5 min in the microwave (700 W). Following, the tissues were delimited using a clear nail polish and blocked using a 4% BSA blocking solution before incubation with the primary StarD13 antibody for 2 h. After washing the slides with ice-cold PBS1X, the tissues were incubated with the appropriate secondary antibody coupled to Alexa fluor-488 fluorophore for 30 min before mounted using a mounting solution mixed with DAPI. Fluorescent cell images were taken using the 20X objective lens of the fluorescent Zeiss Observer Z1 microscope operated by the Zen software (Oberkochen, Germany).

### 2.6. Cell transfection with plasmids and small interfering RNA

SKOV3, Caov-3 and PA-1 ovarian cancer cells were transfected with 10 nM of control Luciferase siRNA or RhoA siRNA, Cdc42 siRNA, Rac1 siRNA and StarD13 siRNA, alone or in combination, using the Hiperfect transfection reagent. Where indicated, 48 h after transfection with the siRNA, the cells were co-transfected with 5 μg of the RhoA or Cdc42 biosensors or with 5 μg of the constitutively active RhoA construct. All assays were performed 72 h after transfection with the siRNA.

### 2.7. Western blot

Proteins extracted from ovarian cancer cells by scraping with Laemmeli sample buffer, were run by SDS-PAGE under standard conditions as previously described (Al Haddad et al., 2020; Al Hassan et al., 2018; Nicolas et al., 2019). After transfer onto a PVDF membrane, the proteins were blocked with 5% bovine serum albumin solution for 1 h, before incubating with the different primary antibodies overnight at 4 °C. Following, the membranes were washed and immunoblotted with the appropriate secondary antibodies for 1 h at room temperature. The bands were visualized with an enhanced chemiluminescent reagent and the images were captured with the Chemidoc imaging system. Densitometry analysis of the expression levels of the different proteins was performed in ImageJ software (National Institute of Health, Massachusetts, USA).

### 2.8. Pull down assay

Proteins were extracted from ovarian cancer cells using the cell lysis buffer provided with the RhoA/Rac1/Cdc42 Activation Assay Combo Kit. The cell lysate was divided in two parts: the first part (500 μl) was incubated with GST-RBD beads or GST-PBD-PAK1 beads, respectively. After incubation on a shaker for 1 h at 4 °C, the samples were centrifuged, and the pellet was washed several times before resuspension in Laemelli sample buffer as described previously (Al Haddad et al., 2020; El Atat et al., 2019; Khalil et al., 2014). The second part, which was not incubated with the beads, was mixed with Laemelli buffer and used as a loading control for RhoA (total RhoA), Rac1 (total Rac1) or Cdc42 (total Cdc42). All proteins, namely, total RhoA, total Rac1, total Cdc42 as well as GTP-RhoA, GTP-Rac1 and GTP-Cdc2 were boiled 5 min at 100 °C before separation by SDS-PAGE as described earlier.

### 2.9. Cell proliferation assay

Ovarian cancer cell Proliferation was determined by adding 10 μl of the WST-1 reagent to control and transfected ovarian cancer cells which were plated in the different wells of a 96 well plate (dilution of WST-1 to media 1:10). The cells were then incubated with WST-1 for 2 h in the incubator (37 °C and 5% CO_2_). Cell proliferation was finally quantified at 450 nm using the Varioskan microplate reader from ThermoFisher scientific (Massachusetts, USA).

### 2.10. Wound healing assay

Control and transfected ovarian cancer cells were grown to confluency before making a wound in the cell monolayer using a sterile pipette tip. The media was then discarded and replaced with serum-free fresh media. Wound healing in control and transfected ovarian cancer cells was monitored by capturing phase-contrast images of the same wound area at t = 0 and t = 48 h post-wounding, using the 10X objective of the Leica inverted microscope. The rate of wound closure (μm/h) presented in the figures was obtained in ImageJ by measuring the distance between the cells at 11 different points of the wound region, and calculating and averaging the speed of wound closure by dividing the distance over time (48 h).

### 2.11. Random cell motility

Random cell motility assay was performed as previously described (Al-Dimassi et al., 2016; Al Haddad et al., 2020; Al Hassan et al., 2018; Hanna et al., 2014a; Khalil et al., 2014; Nasrallah et al., 2014). Briefly, control and transfected ovarian cancer cells plated in 3.5 cm plates were monitored randomly moving in a controlled environment (37 °C and 5% CO_2_). Phase-contrast images of the randomly moving cells were collected every 60 s for 120 min using the 20X objective of the Zeiss Observer Z1 microscope. The total distance traveled by at least 50 randomly moving cells was determined using the ROI tracker in ImageJ. To obtain the average speed of migration in μm/min, the distance was divided over time.

### 2.12. Adhesion assay

Cell adhesion was measured as previously described. Briefly, the wells of a 96-well plate were coated with collagen type I overnight, before washing with washing buffer (0.1% BSA in DMEM media) and blocking in 0.5% BSA blocking solution in the incubator for 1 h. After that, 50 μl of control or transfected ovarian cancer cells suspension containing 4 × 10^5^ cells/ml were added to the wells before placing the plates back in the incubator 30 min. Non-adherent cells were then removed by washing the wells 3 times. Adherent cells in the wells were fixed with 4% paraformaldehyde solution for 10 min, before staining with crystal violet (5 mg/ml in 2% ethanol) for 10 min. Finally, the plates were thoroughly washed and dried before solubilizing crystal violet in DMSO for 30 min. Cell adhesion was quantified at 550 nm using the Varioskan microplate reader from ThermoFisher scientific (Massachusetts, USA).

### 2.13. Invasion assay

Invasion assay was performed as previously described using the collagen-based invasion assay kit from Millipore (Burlington, MA) (Al-Koussa et al., 2020b; Al Haddad et al., 2020; Al Hassan et al., 2018). Briefly, control and transfected ovarian cancer cells were starved for 24 h before resuspension in serum-free quenching medium and plating onto the hydrated inserts. The cells were then placed in wells containing complete medium (10% FBS) and incubated for 24 h. Following, the cells at the bottom surface of the inserts were stained with 400 μl of cell stain for 20 min at room temperature. After extracting the stain with the extraction buffer, 100 μl of the extracted stain were transferred to the wells of a 96-well plate. Finally, the optical density of each sample was measured at 560 nm using the Varioskan microplate reader from ThermoFisher scientific (Massachusetts, USA).

### 2.14. Invadopodia assay

The invasion of control and transfected ovarian cancer cells was examined using the QCM Gelatin Invadopodia Assay kit from Millipore (Massachusetts, USA). Briefly, ovarian cancer cells were plated on fluorescently labeled gelatin matrix for 24 h before fixing the cells with 4% paraformaldehyde solution for 10 min at 37°C, and permeabilizing them with 0.5% Triton-X 100 for 15 min on ice. All cells were blocked in 1% BSA solution for 1 h, before incubation with cortactin or TKS4 primary antibodies overnight at 4°C, and with the appropriate fluorophore-conjugated secondary antibodies for 1 h. The cells were imaged using a 63X objective lens on Zeiss Observer Z1 fluorescent microscope and the degradation of the matrix was measured upon quantification of dark areas lacking cortactin or TKS4 signaling using ImageJ.

### 2.15. FRET imaging and analysis

FRET analysis was performed as previously described (Al Haddad et al., 2020). Briefly, ovarian cancer cells were transfected with 2.5 μg of RhoA fluorescence resonance energy transfer 2 (FRET)-based biosensor (Pertz et al., 2006) or Cdc42-based FRET biosensor (Hanna et al., 2014b) using lipofectamine. After 24 h, the cells were imaged in CFP, YFP, FRET and DIC channels using the 63X objective lens of the Zeiss Observer Z1 fluorescent microscope (Oberkochen, Germany). YFP exciter and emitter were S500/20 and S535/30 (YFP/acceptor image), respectively. CFP exciter and emitter were S430/25 and S470/30 (CFP/donor image) or S535/30 for FRET image (Al Haddad et al., 2020). For FRET analysis, the background was flatfield corrected and subtracted from the signal and a threshold was applied to the YFP images to obtain a binary mask with values of 1 for the area inside the cell and 0 for the background. Next, the background was removed from the ratio calculations by multiplying the CFP and FRET images by the mask. RhoA and Cdc42 activation were calculated by dividing the FRET image over the donor image. Finally, FRET signals were quantified by averaging the mean FRET ratio in all the cell area, normalizing the values to control cells (untreated) and expressing the difference as fold change as described previously (Al Haddad et al., 2020; Hodgson et al., 2010). All single cell analysis experiments are performed as three independent experiments and images of 15 cells from every experiment collected (data is average from 45 cells).

### 2.16. Quantification of focal adhesions

The area and number of focal adhesion were quantified in ImageJ using the CLAHE and Log3D plugins as described previously (Al-Koussa et al., 2019; Al Haddad et al., 2020; Horzum et al., 2014). CLAHE enhances the local contrast of the image and Log3D filters the image based on predefined parameters for focal adhesions detection and analysis (Al Haddad et al., 2020; Horzum et al., 2014). The area of focal adhesions observed upon staining with vinculin was expressed in arbitrary unit (a. u.). The number of focal adhesion was presented as absolute values of the means for each condition.

### 2.17. Statistical analysis

The results reported represent average values from three independent experiments. The error estimates are given as ± SEM. The *p*-values were calculated by two way ANOVA or *t*-test to check if the changes observed in the results were significant.

## 3. Results

### 3.1. StarD13 is a potential tumor suppressor in ovarian cancer

**Fig. 1.**
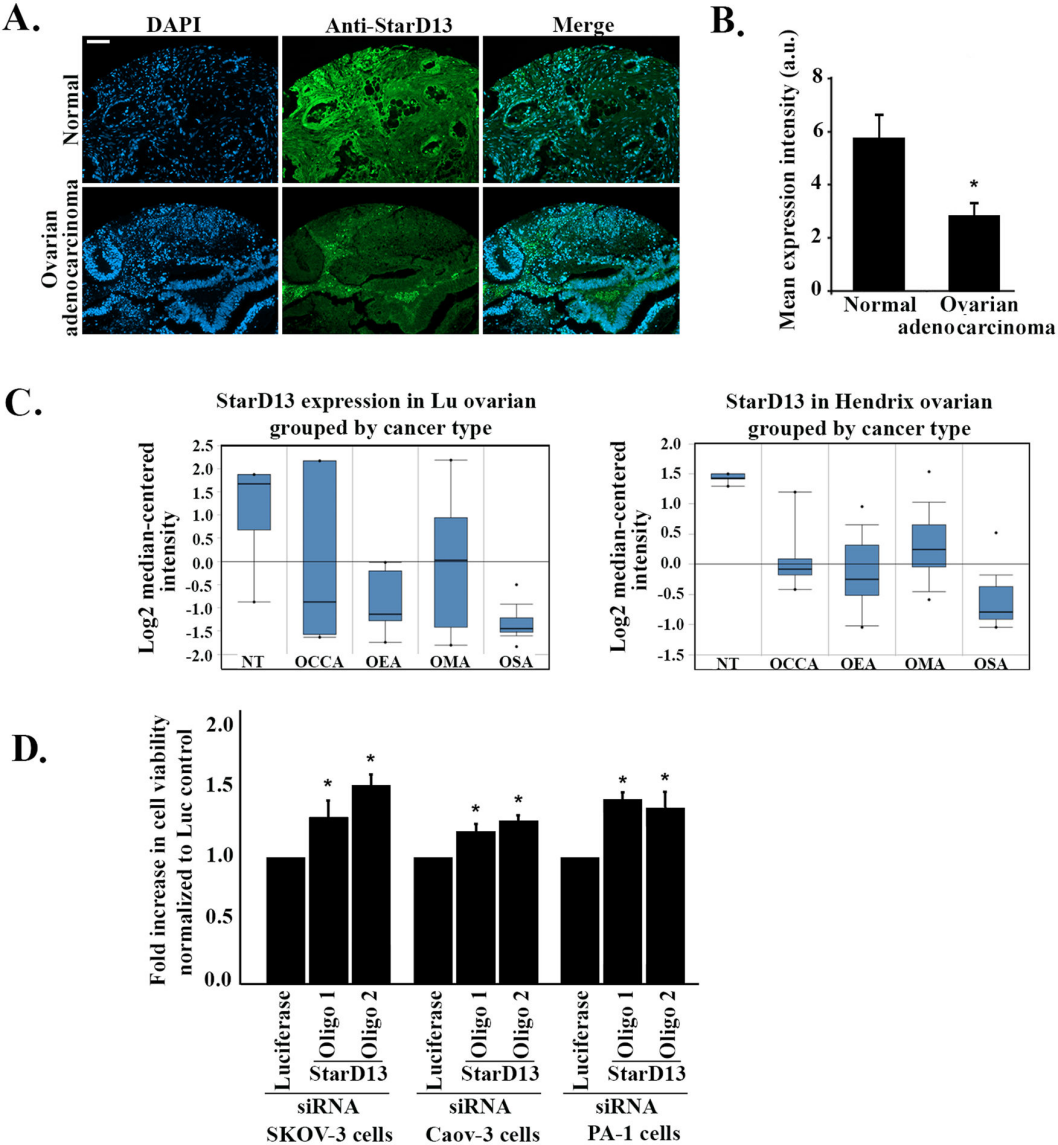
StarD13 is a potential tumor suppressor in ovarian cancer. A. Normal ovarian and ovarian adenocarcinoma tissue samples were stained against StarD13. The micrographs show STARD13 expression in green, DAPI expression in blue and the merged images. The scale bar is 100 μm. **B.** StarD13 expression levels in normal and ovarian adenocarcinoma tissues was quantified in imageJ and expressed as mean expression intensity. **C.** StarD13 mRNA samples were analyzed from Oncomine data sets and the results were plotted using normal versus different ovarian cancer types. NT: Non tumorigenic, OCCA: ovarian clear cell adenocarcinoma, OEA: ovarian endometrioid adenocarcinoma, OMA: ovarian mucinous adenocarcinoma, OSA: ovarian serous adenocarcinoma. **D.** Fold increase in cell proliferation of Luciferase siRNA and StarD13 siRNA (oligo 1 and oligo 2) transfected SKOV-3, Caov-3 and PA-1 ovarian cancer cells was normalized to the luciferase control. The data are representative of the mean ± SEM of three independent experiments. Significance was set at p < 0.05.

To understand the role of StarD13 in ovarian cancer cells, we determined the expression profile of StarD13 in normal ovarian tissues as well as in tissues obtained from patients with ovarian adenocarcinoma (Fig. 1A). Immunohistochemistry analysis showed a significant 2-fold decrease in the expression levels of StarD13 in ovarian adenocarcinoma biopsies as compared to normal ones (Fig. 1B). This was further confirmed by mining the Oncomine database for microarray analysis of StarD13 expression in different ovarian cancer types from two datasets. StarD13 expression levels were significantly lower in almost all ovarian cancer types investigated relative to the normal tissues, thus suggesting a potential tumor suppressor role for StarD13 in ovarian cancers (Fig. 1C). To further test this hypothesis, we first knocked down StarD13 in three different ovarian cancer cell lines: SKOV-3 (Supplemental Fig. S1A), Caov-3 (Supplemental Fig. SIB) and PA-1 (Supplemental Fig. SIC) then examined the impact of this knock down on cell proliferation. This was achieved by transfecting cells with two different StarD13 specific siRNA oligos (oligo 1 and oligo 2). Supplemental Fig. S1 demonstrates that both oligos efficiently decreased StarD13 expression levels as compared to controls. Specifically, StarD13 expression was reduced by around 80% in SKOV-3 and PA-1 cell lines and around 50% in Caov-3 cell line as compared to the luciferase control (Supplemental Fig. S1D). Proliferation of Caov-3 cells depleted of StarD13 increased by around 25%, compared to controls, while proliferation of SKOV-3 and PA-1 cells depleted of StarD13 increased by around 50% compared to controls (Fig. 1D), further demonstrating a potential tumor suppressor role played by StarD13 in ovarian cancer cells as previously observed in other tumor types (Leung et al., 2005).

### 3.2. StarD13 depletion does not affect the 2D migration of ovarian cancer cells

**Fig. 2.**
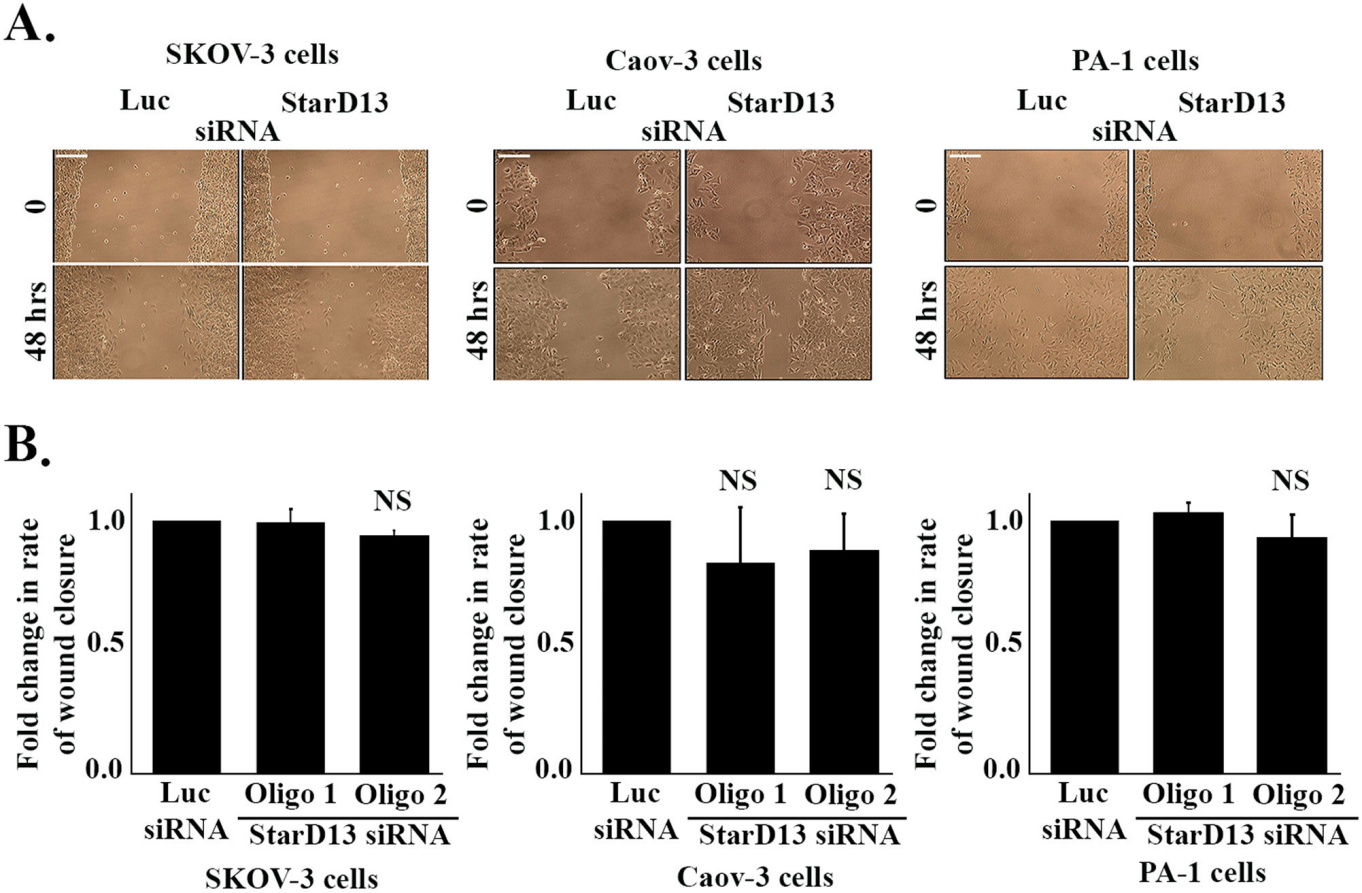
StarD13 depletion does not affect the 2D migration of ovarian cancer cells. A. SKOV-3, Caov-3 and PA-1 ovarian cancer cells were transfected with Luciferase siRNA, or StarD13 siRNA for 72 h before creating a wound in the cell monolayer. Wound images of the same frame were taken at t = 0 (left panel) and t = 48 h after wounding (right panel). B. Wound closure rate of Luciferase siRNA and StarD13 siRNA transfected (Oligo 1 and Oligo 2) SKOV-3 (left panel), Caov-3 (middle panel) and PA-1 (right panel) ovarian cancer cells was quantified in imageJ, and expressed as fold change compared to the Luciferase siRNA control. The scale bar is 100 μm. The data are representative of the mean ± SEM of three independent experiments. Significance was set at p < 0.05.

After having established the anti-proliferative potential of StarD13 in ovarian cancer cells, we investigated its ability to modulate cell motility using two approaches: wound healing and time-lapse assays. The results show similar wound closure areas in control and StarD13-depleted SKOV-3, Caov-3 and PA-1 cells (Fig. 2A). Quantitatively, StarD13 knock down did not affect the rate of wound closure in any of these cell lines either (Fig. 2B). The time lapse assays performed on control and StarD13-depleted SKOV-3 and Caov-3 also support these conclusions whereby both control and SKOV-3 depleted cells exhibit similar cell speed as the luciferase control (Table 1). This data potentially exclude a role for StarD13 in regulating the migration of ovarian cancer cells in 2D.

This was surprising since previous reports from studies performed in astrocytoma, breast cancer, colon cancer and lung cancer, showed that StarD13 depletion inhibited cancer cell migration due to the dysregulation of RhoA activation (Al Haddad et al., 2020; Hanna et al., 2014a; Khalil et al., 2014; Nasrallah et al., 2014). Indeed in these cells, the dysregulation of RhoA led to a decrease in cell migration (Supplemental Fig. S2). When RhoA was depleted in SKOV-3 and Caov-3 (The western blot results presented in Supplemental Fig. S2 show significant reduction of RhoA expression in both SKOV-3 and Caov-3 cells) upon transfection with the RhoA siRNA or overexpressed in the constitutively active form (cells transfected with RhoA-CA), both cells showed a decrease in cell motility (Supplemental Fig. S2B and S2D and Supplemental movie S1). This shows that the regulation of activation/inactivation cycling of RhoA is also needed in ovarian cancer cells for effective cell migration, however, this regulation seems to be StarD13-independent.

Supplementary material related to this article can be found online at doi: 10.1016/j.ejcb.2021.151197.

### 3.3. StarD13 is a GAP for Cdc42 in ovarian cancer cells

**Fig. 3.**
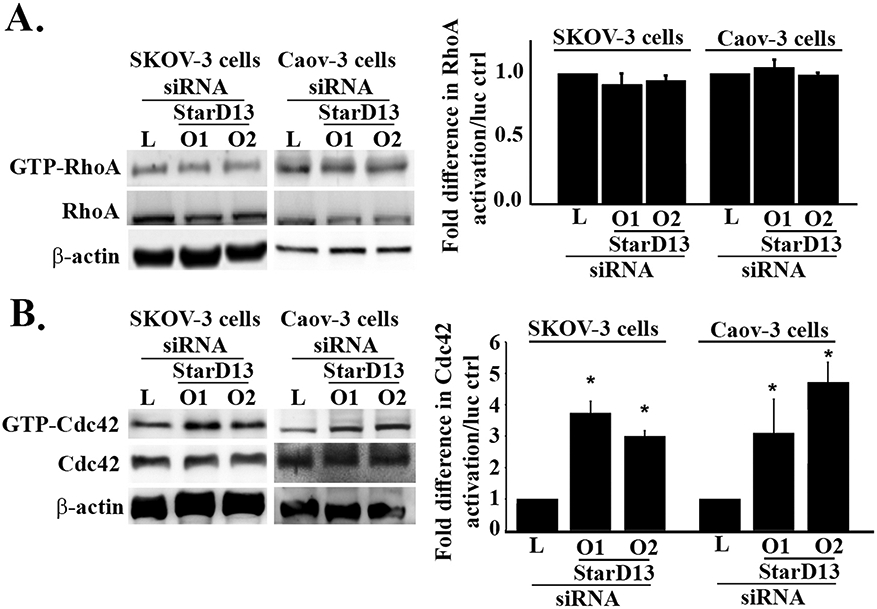
Depletion of StarD13 leads to an increase in GTP-Cdc42 but not GTP-RhoA in ovarian cancer cells. A. Left panel: Cell lysates of Luciferase siRNA and StarD13 siRNA (oligo 1 and oligo 2) transfected SKOV-3 and Caov-3 cancer cells were incubated with or without GST-RBD beads and blotted against RhoA. Right panel: Bar graph illustrating the quantification of GTP-RhoA/RhoA in SKOV-3 and Caov-3 cells expressed as fold difference in GTP-RhoA/RhoA of StarD13 siRNA transfected ovarian cancer cells relative to the luciferase control. B. Left panel: Cell lysates of Luciferase siRNA and StarD13 siRNA (oligo 1 and oligo 2) transfected SKOV-3 and Caov-3 cancer cells were incubated with or without GST-PBD beads and blotted against Cdc42. Right panel: Bar graph illustrating the quantification of GTP-Cdc42/Cdc42 in SKOV-3 and Caov-3 cells expressed as fold difference in GTP-Cdc42/Cdc42 of StarD13 siRNA transfected ovarian cancer cells relative to the luciferase control. The data are representative of the mean ± SEM of three independent experiments. Significance was set at p < 0.05.

To confirm the GAP function of StarD13, we performed a pull-down assay to investigate the differences in GTP loading of RhoA and Cdc42 in SKOV-3 and Caov-3 cancer cells in the presence or absence of StarD13. Western blot analysis revealed an approximate 3-fold increase in GTP-Cdc42 levels in both SKOV-3 and Caov-3 cells upon StarD13 depletion, compared to controls (Fig. 3B). This strongly suggests that StarD13 is a GAP for Cdc42 in ovarian cancer as it was shown in other tumor types (Ching et al., 2003; Kawai et al., 2007; Wong et al., 2003). On the other hand, Fig. 3A reveals that GTP-RhoA levels were not affected by the depletion of StarD13 in both cell lines. This could explain why RhoA affects cell migration independently of StraD13, as described above, in these cells.

### 3.4. StarD13 attenuates ovarian cancer cells adhesion through the inhibition of Cdc42/Rac1

**Fig. 4.**
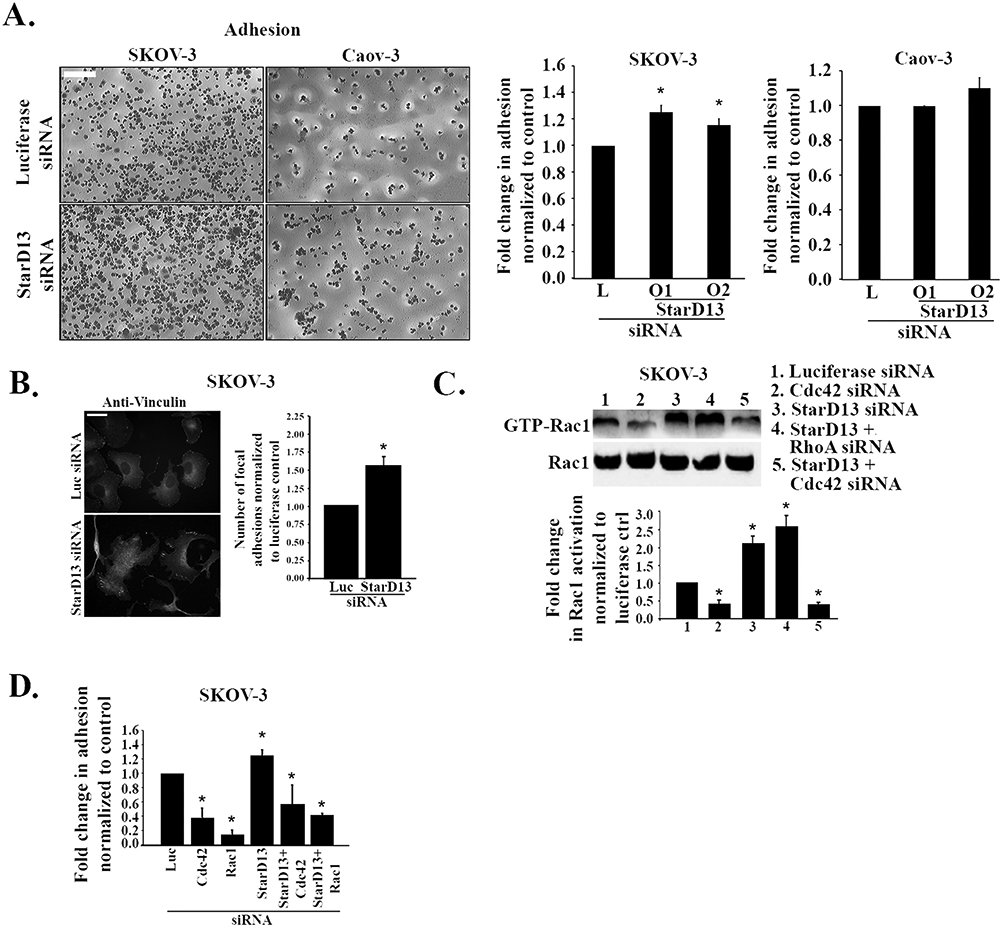
Rac1 mediates StarD13 inhibition of ovarian cancer cells adhesion. A. Left panel: Representative micrographs of crystal violet stained Luciferase siRNA or StarD13 siRNA transfected SKOV-3 and Caov-3 cells. The scale bar is 100 μm. Right panel: Bar graphs illustrating the fold change in adhesion of Luciferase siRNA or StarD13 siRNA transfected SKOV-3 and Caov-3 cells measured by ELISA at 560 nm. B. Right panels: Micrographs of SKOV-3 cells transfected with Luciferase siRNA or StarD13 siRNA and immunostained against vinculin (green). The scale bar is 10 μm. Right panel: Bar graph illustrating the quantification of the number of focal adhesion in SKOV-3 cells transfected with Luciferase siRNA or StarD13 siRNA. C. Cell lysates of SKOV3 cells transfected with Luciferase siRNA, StarD13 siRNA, Cdc42 siRNA, StarD13 siRNA in combination with Cdc42 siRNA or RhoA siRNA were incubated with or without GST-PBD beads and blotted against Rac1. Right panel: Bar graph illustrating the quantification of GTP-Rac1/Rac1 in SKOV-3 cells as fold difference in GTP-Rac1/Rac1 normalized to the luciferase control. D. Bar graph illustrating the fold change in adhesion of SKOV-3 cells transfected with Luciferase siRNA, StarD13 siRNA, Cdc42 siRNA, Rac1 siRNA or StarD13 siRNA in combination with Cdc42 siRNA and Rac1 siRNA, respectively. The data are representative of the mean ± SEM of three independent experiments. Significance was set at p < 0.05.

Previous studies from our laboratory and others reported an effect of StarD13 on cell adhesion and a potential localization of StarD13 to focal adhesions (Al Haddad et al., 2020; Hanna et al., 2014a; Holeiter et al., 2008; Kawai et al., 2007, 2009; Khalil et al., 2014). Here, we investigate the effects of StarD13 knock down on the adhesion of SKOV-3 and Caov-3 cells to the extra cellular matrix. Fig. 4A reveals a 40% and 20% increase in cellular adhesion to collagen of SKOV-3 and Caov-3 cells transfected with StarD13 siRNA, respectively, as compared to controls. Immunostaining for focal adhesion using anti-vinculin in control and StarD13-depleted SKOV-3 cells further confirmed the role of StarD13 in ovarian cancer cell adhesion to the matrix. Indeed, the silencing of StarD13 triggered a 60% increase in the number of focal adhesions in StarD13 siRNA transfected cells as compared to the luciferase control (Fig. 4B). Previously, we had also established that the effect of StarD13 depletion on adhesion to be mediated through a lack of RhoA inactivation (Al Haddad et al., 2020; Hanna et al., 2014a; Khalil et al., 2014). Having established in ovarian cancer that StarD13 has only Cdc42 as a downstream target, we tested the possibility that the increase in adhesion in the Stard13-depleted cells is mediated through the absence of Cdc42 inactivation, which leads to an increase in Rac1 activation. We knocked down Cdc42 in SKOV-3 and Caov-3 cells using sequence specific siRNA targeted against Cdc42 (Cdc42 siRNA oligo 1 and oligo 2). Supplemental Fig. S3A shows that Cdc42 siRNA oligo 1 and oligo 2 efficiently decreased the expression level of Cdc42 in SKOV-3 and Caov-3 cancer cells by 80% and 60%, respectively as compared to the control. As expected, Cdc42 depletion led to a decrease in levels of GTP-Rac1 in SKOV-3 cells (Fig. 4C). In addition, StarD13 depletion led to an increase in GTP-Rac1 levels which was reversed in the Cdc42 double knock down but not in the RhoA double knock down (Fig. 4C). This potentially suggests that Cdc42 is upstream of Rac. Hence, the depletion of StarD13 might relieve the Cdc42 inhibition, potentially leading to GTP-Rac1 accumulation. Having established the increase in GTP-Rac1 in response to StarD13 depletion, we then directly tested the involvement of the Rac1 Rho GTPase in mediating the increase in cellular adhesion upon StarD13 depletion. To this aim, we effectively knocked down Rac1 (by approximately 80%) (Supplemental Fig. S3B) and assessed the effects of StarD13, Rac1, Cdc42, StarD13 + Cdc42 or StarD13 + Rac1 depletion on SKOV-3 cell adhesion. In contrast to StarD13 depletion, which increased cell adhesion by 40% as compared to the control, both Cdc42 and Rac1 depletions decreased SKOV-3 cell adhesion to collagen by 60% and 80%, respectively. Knocking down StarD13 with either Cdc42 or Rac1 countered StarD13 depletion effects and decreased cell adhesion as compared to the control (Fig. 4D). Collectively, this suggests that StarD13 attenuates adhesion through the inhibition of Cdc42, which in turn leads to the inhibition of Rac1.

### 3.5. Cdc42 mediates inhibition of ovarian cancer cell protrusions by StarD13

**Fig. 5.**
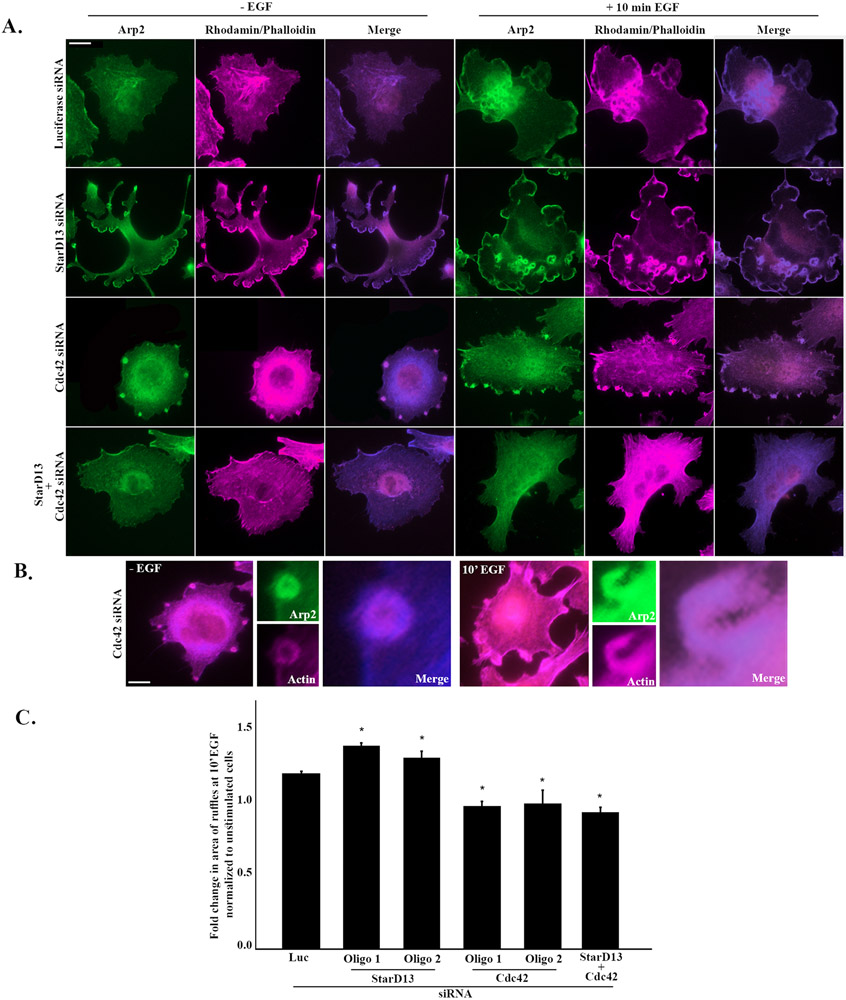
Cdc42 mediates StarD13 inhibition of ovarian cancer cell protrusions. A. SKOV-3 cells transfected with Luciferase siRNA, StarD13 siRNA, Cdc42 siRNA or StarD13 siRNA in combination with Cdc42 siRNA, respectively, were either starved or stimulated with EGF for 10 min. All cells were then immunostained against Arp-2 (green) and rhodamine-phalloidin (magenta) and imaged using the 63x lens of the Zeiss fluorescent microscope. The scale bar is 10 μm. B. SKOV-3 cells were transfected with Cdc42 siRNA and stimulated or not with EGF for 10 min before immunostaining against Arp-2 (green) and rhodamine-phalloidin (magenta). The merged images and magnification of the Arp-2 and actin pockets are also shown. The scale bar is 10 μm. C. In order to detect the ruffles, cells were threshholded in the Arp-2 channel and ruffles were selected and area calculated. The bar graph illustrating the fold change in the area of ruffles of EGF stimulated SKOV-3 cells transfected with Luciferase siRNA, StarD13 siRNA, Cdc42 siRNA or StarD13 siRNA in combination with Cdc42 siRNA normalized to the unstimulated control. The data are representative of the mean ± SEM of three independent experiments. Significance was set at p < 0.05.

Knowing that Cdc42 is the main effector of StarD13 in these cells, we then looked at the effect of StarD13 depletion on cell protrusion, a main event during cancer migration regulated by Cdc42 (El-Sibai et al., 2007). In order to stimulate pronounced protrusions in these cells, we stimulated the cells with the epidermal growth factor (EGF), a known chemoattractant of ovarian cancer cells (Alper et al., 2001; Hudson et al., 2009; Moss et al., 2009). SKOV-3 cells were transfected with Luciferase siRNA, StarD13 siRNA, Cdc42 siRNA, or StarD13 siRNA in combination with Cdc42 siRNA. The cells were then starved and stimulated with EGF before staining with rhodamine-phalloidin and Arp2 to mark actin rich protrusions and subsequently quantify the area of membrane ruffles/protrusions. Starved and EGF stimulated SKOV-3 cells depleted of StarD13 exhibited an increase in dorsal membrane ruffles and protrusions as well as a loss of directionality of these structures as compared to the control (Fig. 5A and Supplemental movie S2). Moreover, StarD13-depleted cells showed a higher increase in protrusion and ruffles in response to EGF stimulation. Depletion of Cdc42 in combination with StarD13 eliminated the phenotype observed in both starved and EGF-stimulated cells (Fig. 5A and Supplemental movie S2). Interestingly, Fig. 5B shows that even though Cdc42 depleted SKOV-3 cells lack protrusion and membrane ruffles, the cells show “pockets” of Arp2 and actin accumulation. This suggests that in the absence of Cdc42, the actin nucleator Arp2/3 does not distribute properly for the protrusions to mature and branch. Quantitatively, the protrusion/ruffle area of EGF-stimulated SKOV-3 cells depleted of StarD13 increased by 30% as compared to the control (Fig. 5C). The results also show that Cdc42 depleted SKOV3 cells fold change in ruffle area was reduced by 15% as compared to the control and by 20% for StarD13 and Cdc42 depleted SKOV3 cells (Fig. 5C).

Supplementary material related to this article can be found online at doi: 10.1016/j.ejcb.2021.151197.

### 3.6. Cdc42 mediates StarD13 inhibition of ovarian cancer cell invasion

**Fig. 6.**
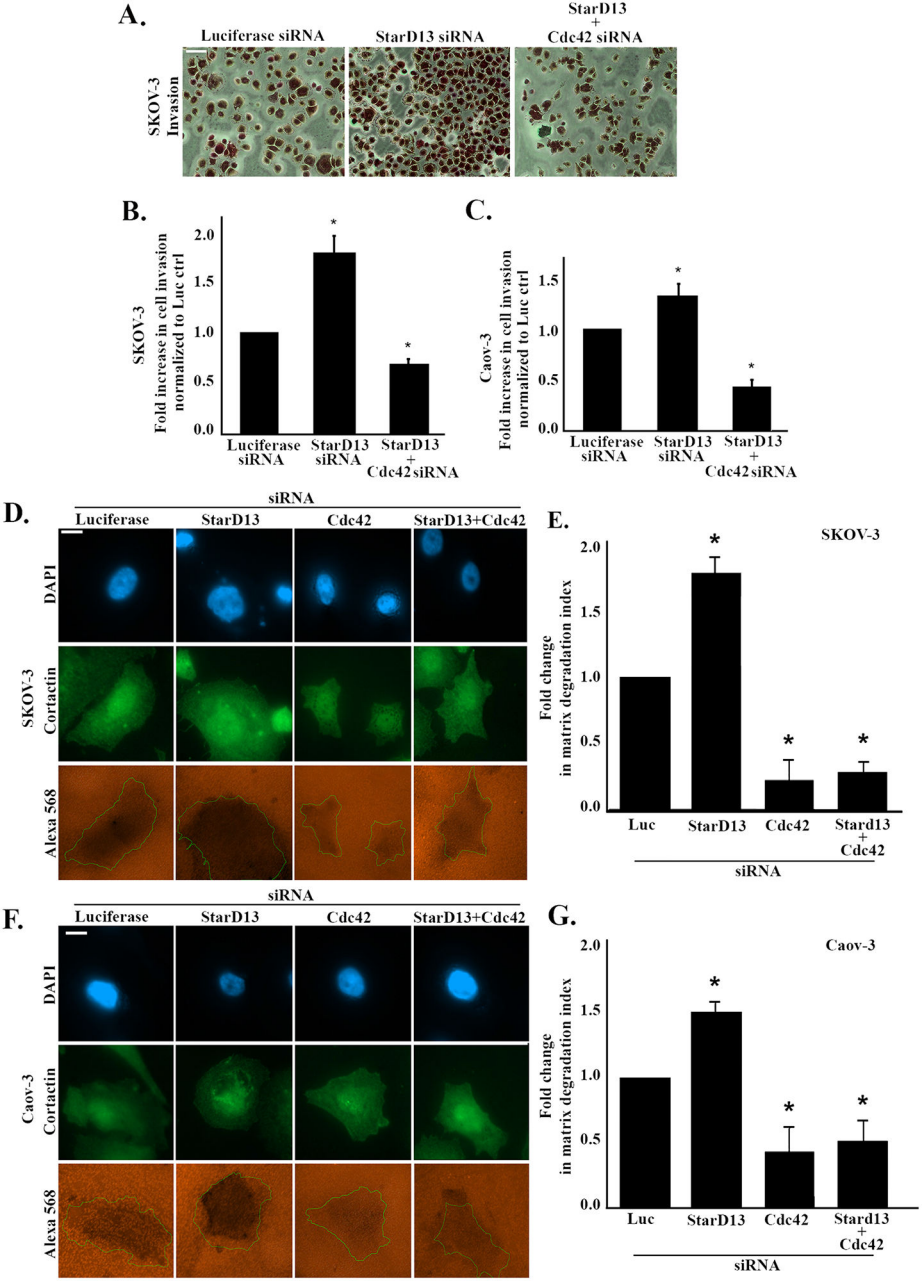
StarD13 inhibits invasion and matrix degradation in ovarian cancer cells by inhibiting Cdc42. A. Representative micrographs of SKOV-3 transfected with Luciferase siRNA or StarD13 siRNA alone or in combination with Cdc42 siRNA, and allowed to invade the bottom of collagen-coated membranes towards 10% FBS. Scale bar is 100 μm. B. Quantification of the fold increase in invaded SKOV-3 cells normalized to the luciferase control. C. Quantification of the fold increase in invaded Caov-3 cells normalized to the luciferase control. The data are representative of the mean ± SEM of three independent experiments. Significance was set at p < 0.05. D. SKOV-3 cells were transfected with Luciferase siRNA, StarD13 siRNA, Cdc42 siRNA or StarD13 siRNA in combination with Cdc42 siRNA, respectively, before plating on fluorescently labeled gelatin. Following, the cells were stained against cortactin to mark invadopodia. The micrographs show the change in invaded area (orange), as well as the intensities of cortactin (green), and DAPI (blue). Images were visualized using the 63x objective of the Zeiss fluorescent microscope. Scale bar is 10 μm. E. Quantification of the area invaded by SKOV-3 cells was performed in imageJ and expressed as fold change in matrix degradation index. F. Caov-3 cells were transfected with Luciferase siRNA, StarD13 siRNA, Cdc42 siRNA or StarD13 siRNA in combination with Cdc42 siRNA, respectively, before plating on fluorescently labeled gelatin. Following, the cells were stained against cortactin to mark invadopodia. The micrographs show the change in invaded area (orange), as well as the intensities of cortactin (green), and DAPI (blue). Images were visualized using the 63x objective of the Zeiss fluorescent microscope. Scale bar is 10 μm. **G.** Quantification of the area invaded by Caov-3 cells was performed in imageJ and expressed as fold change in matrix degradation index. The data are representative of the mean ± SEM of three independent experiments. Significance was set at p < 0.05.

Cdc42 is directly implicated in the regulation of cancer cell invasion, therefore we investigated the role of StarD13 in regulating the invasion of ovarian cancer cells (Al-Koussa et al., 2020a; Al Haddad et al., 2020; Evers et al., 2000; Fortin Ensign et al., 2013; Keely et al., 1997; Kwiatkowska et al., 2012; Yang et al., 2019b). Using transwell inserts, we tested the ability of control, StarD13 siRNA or StarD13 siRNA + Cdc42 siRNA transfected SKOV-3 or Caov-3 cells to invade in vitro. The micrographs presented in Fig. 6A reveal an increased number of SKOV-3 invaded cells in response to the depletion of StarD13. However, fewer invading cells were observed when Cdc42 is depleted in combination with StarD13 (Fig. 6A). Quantitatively, SKOV3 and Caov-3 cells depleted of StarD13 exhibited 80% and 30% increase in invasion, respectively as compared to the control (Figs. 6B and 6C). However, Cdc42 depletion was able to reverse the effect of StarD13 depletion in SKOV-3 and Caov-3 cancer cells and reduce the number of invaded cells by around 50% as compared to the StarD13 depleted cells.

### 3.7. StarD13 inhibits matrix degradation in ovarian cancer cells by inhibiting Cdc42

Matrix degradation is a critical step in cancer invasion and metastasis. We therefore next tested the ability of StarD13 to modulate ovarian cancer cell matrix degradation using the gelatin invadopodia assay. StarD13 and Cdc42 were silenced alone and in combination cells before plating SKOV-3 and Caov-3 cells on fluorescently labeled gelatin matrix for 24 h and subsequent staining with cortactin to the mark invadopodia. Fluorescent micrographs show that StarD13 depletion increases the matrix degraded area in SKOV-3 and Caov-3 cells (Figs. 6D and 6F). Cdc42 depletion alone or in combination with StarD13 decreases matrix degradation in both cell lines as compared to the control. Quantitatively, StarD13 depletion increased the degraded area by around 70% and 50% in SKOV-3 and Caov-3, respectively as compared to the control (Figs. 6E and 6G). Cdc42 depletion alone or in combination with StarD13 reduces matrix degradation by around 80% and 60% respectively, in SKOV-3 and Caov-3 cells as compared to the control (Figs. 6E and 6G).

### 3.8. StarD13 inhibits potential invadopodia formation in ovarian cancer cells by inhibiting Cdc42

**Fig. 7.**
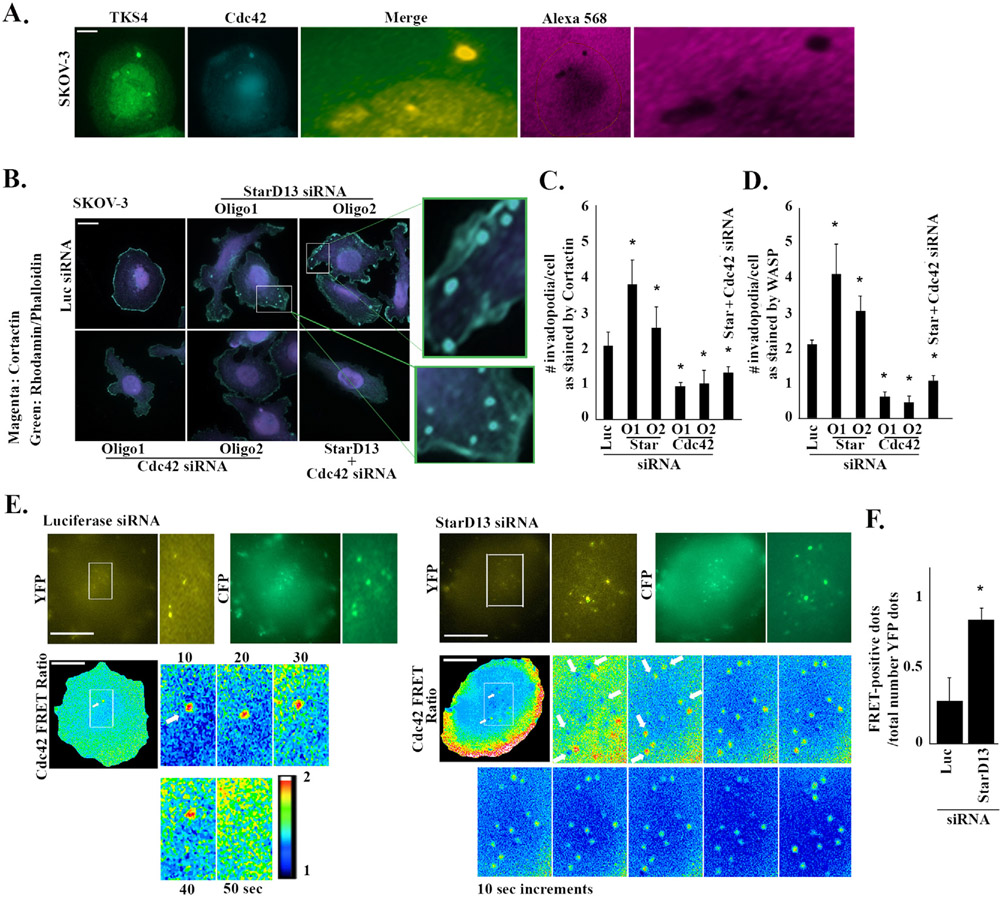
StarD13 inhibits the formation of invadopodia-like structures in ovarian cancer cells by inhibiting Cdc42. A. SKOV-3 cells were plated on fluorescently labeled gelatin (Alexa568) before staining against Cdc42 and TKS-4 to mark invadopodia. The micrographs show the change in invaded area (magenta), the intensities of TKS-4 (green), and Cdc42 (blue) as well as the merged image. Scale bar is 10 μm. B. SKOV-3 cells transfected with Luciferase siRNA, StarD13 siRNA (oligo 1 and oligo 2), Cdc42 siRNA (oligo 1 and oligo 2) or StarD13 siRNA in combination with Cdc42 siRNA, respectively. All cells were then immunostained against cortactin (green) and rhodamine-phalloidin (magenta) and imaged using the 63x lens of the Zeiss fluorescent microscope. The magnified frames are pointed out on the original images as white boxes. Scale bar is 10 μm. C. Bar graph illustrating the quantification of the number of invadopodia (seen by cortactin staining) per cell for SKOV-3 cells transfected with Luciferase siRNA, StarD13 siRNA (oligo 1 and oligo 2), Cdc42 siRNA (oligo 1 and oligo 2) or StarD13 siRNA in combination with Cdc42 siRNA. D. Bar graph illustrating the quantification of the number of invadopodia (seen by WASP staining) per cell for SKOV-3 cells transfected with Luciferase siRNA, StarD13 siRNA (oligo 1 and oligo 2), Cdc42 siRNA (oligo 1 and oligo 2) or StarD13 siRNA in combination with Cdc42 siRNA. E. SKOV3 cells were transfected with Luciferase siRNA or StarD13 siRNA for 72 h before transfecting with the Cdc42 FRET biosensor. The cells were then imaged in CFP, YFP and FRET channels. Upper panels: Representative micrographs of SKOV-3 cells imaged in the different channels. Lower panels: Ratiometric images for Cdc42 FRET ratio were obtained by normalizing the raw FRET images to the CFP images as described in the materials and methods. F. Bar graph illustrating the quantification of the FRET signal (Ratio of FRET/YFP signal) in the total cell area. The data are representative of the mean ± SEM of three independent experiments. Significance was set at p < 0.05.

In a recent study, we showed that Cdc42 localizes to TKS4-labeled invadopodia and to the sites of matrix degradation in lung cancer cells. We also showed that StarD13 depletion leads to an increase in these potential invadopodial structures and that this is mediated through an increase in GTP-Cdc42 levels (Al Haddad et al., 2020). In addition, Cdc42 is a known component and regulator of invadopodia (Murphy and Courtneidge, 2011). To further understand the mechanism of StarD13 regulation of ovarian cancer invasion, we investigated the effects of StarD13 and Cdc42 inhibition on invadopodia formation. In order to correctly detect invadopodia, the cells were immunostained against TKS4, a key and exclusive component of invadopodia (Murphy and Courtneidge, 2011). Fig. 7A shows that Cdc42 co-localizes with TKS4-labeled (mature) invadopodia and the area of matrix degradation. Staining the cells with cortactin (and rhodamine-phalloidin) revealed an increase in the collective number of actin-rich dots in StarD13-depleted SKOV-3 cells (Fig. 7B). In contrast, SKOV-3 cells depleted of Cdc42 (alone or in combination with StarD13) did not display cortactin-stained invadopodia (Fig. 7B). These phenotypes were further confirmed upon staining the cells with either cortactin or WASP and quantitation of the total number of actin-rich dots per cell (Figs. 7C and 7D). The data support that StarD13 knock down increases the potential number of invadopodia as compared to the luciferase control by up to two folds, and that Cdc42 depletion either alone or in combination with StarD13 decreases the potential number of invadopodia by around 1–1.5 fold (Figs. 7C and 7D). After establishing that Cdc42 localizes to TKS5-positive invadopodia, we looked at the localization of GTP-Cdc42 with FRET analysis of control and StarD13-depleted SKOV-3 cells transfected with a Cdc42 activation biosensor (Figs. 7E and 7F and Supplemental movies S3-S6) (Hanna et al., 2014b). StarD13-depleted SKOV-3 cells exhibited a significant 1.5-fold increase in GTP-Cdc42-positive dots (potential invadopodia with Cdc42 activation), as compared to the luciferase control (Figs. 7E and 7F). The data also reveals that all invadopodia in StarD13-depleted SKOV-3 have GTP-Cdc42 (Fig. 7E and Supplemental movies S6-S6). This is possibly due to the exaggerated activation of Cdc42 in response to StarD13 knock down and the persistence of GTP-Cdc42 in invadopodia (Supplemental movie S6). Conversely, the YFP channel in luciferase control SKOV-3 cells transfected with the Cdc42 biosensor display more dots than those seen in the Cdc42 FRET ratio image (Fig. 7E). This would indicate the localization of the Cdc42 biosensor (seen in YFP) but lack of Cdc42 activation (potentially due to the presence of StarD13). Indeed, Fig. 7E and Supplemental movies S3 and S6 further indicate that potential Cdc42 activation signal is lost in invadopodia of Luciferase siRNA control cells after around 1 min, while the invadopodia of SKOV-3 cells depleted of StarD13 persisted as revealed through the Cdc42 FRET signal (Fig. 7E and Supplemental movies S3-S6). Interestingly, the first frames in StarD13-depleted cells show that, at an earlier stage, invadopodia lack GTP-Cdc42 as evidenced by the lowest FRET ratio pointed out with arrows in Fig. 7E, while the following ones reveal dot like spheres with GTP-Cdc42 which is also evidenced by the highest FRET ratio pointed out with the arrows in Fig. 7E (Supplemental movies S3-S6). This is an opportune visualization of Cdc42 localization to the invadopodia (seen as a low FRET signal since YFP signal is high reflecting localization while the biosensor has not activated yet) followed shortly by its activation as the FRET signal increased.

Supplementary material related to this article can be found online at doi: 10.1016/j.ejcb.2021.151197.

Supplementary material related to this article can be found online at doi: 10.1016/j.ejcb.2021.151197.

Supplementary material related to this article can be found online at doi: 10.1016/j.ejcb.2021.151197.

Supplementary material related to this article can be found online at doi: 10.1016/j.ejcb.2021.151197.

### 3.9. RhoA is required for matrix degradation and invasion independently from StarD13

RhoA has been previously shown to regulate matrix degradation through a mechanism that involves delivery of matrix metalloproteinases to the invadopodia (Sakurai-Yageta et al., 2008). While transfecting SKOV-3 cells with a RhoA FRET biosensor (Pertz et al., 2006), we were able to detect a concentration of GTP-RhoA in dots (potential invadopodia) (Supplemental Fig. S4A). Supplemental Fig. S4A and Supplemental movies S7 and S8 illustrate an increase in the number of invadopodia in StarD13-depleted cells as compared to the control (in line with our previous findings) and a localization of GTP-RhoA to the invadopodia in both control and StarD13-depleted cells. This was evidenced by the FRET signal measured in all cell area and per invadopodia where no significant change in levels of GTP-RhoA was detected upon the depletion of StarD13 (Supplemental Fig. S4B and C and Supplemental movies S7 and S8). The movies revealed that RhoA activation potentially persists along with invadopodia presence. RhoA depletion also inhibited gelatin degradation and invasion by 70% and 40% respectively (Supplemental Fig. S4D and E). This data proved that RhoA is required for SKOV-3 cancer cells invasion. Having established no significant effect of StarD13 depletion on levels of GTP-RhoA in these cells, this suggests that RhoA plays a role in invadopodial function and in matrix degradation and invasion independently of Stard13 in ovarian cancer cells.

Supplementary material related to this article can be found online at doi: 10.1016/j.ejcb.2021.151197.

Supplementary material related to this article can be found online at doi: 10.1016/j.ejcb.2021.151197.

### 3.10. StarD13 depletion activates Cdc42 and triggers the maturation of TKS-rich dots

**Fig. 8.**
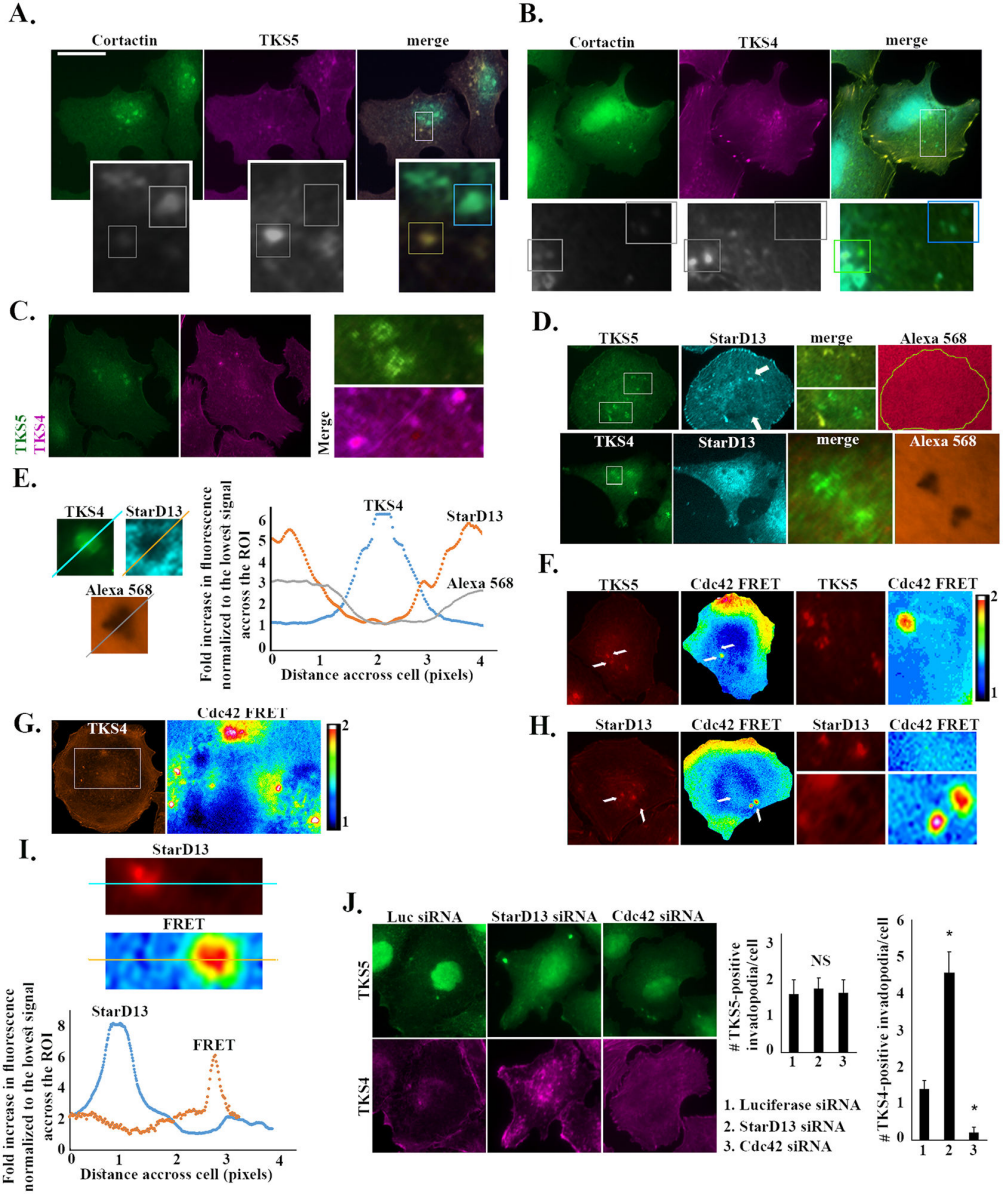
StarD13 depletion activates Cdc42 and triggers the maturation of invadopodia-like structures. A. SKOV-3 cells were immunostained against cortactin (green) and TKS5 (magenta) and imaged using the 63x lens of the Zeiss fluorescent microscope. The magnified frames are pointed out on the original images as white boxes. The scale bar is 10 μm. B. SKOV-3 cells were immunostained against cortactin (green) and TKS4 (magenta) and imaged using the 63x lens of the Zeiss fluorescent microscope. The magnified frames are pointed out on the original images as white boxes. The scale bar is 10 μm. C. SKOV-3 cells were immunostained against TKS5 (green) and TKS4 (magenta) and imaged using the 63x lens of the Zeiss fluorescent microscope. The magnified frames are ROIs (region of interests) from the merged channel of TKS5 and TKS4. Scale bar is 10 μm. D. Upper panels: SKOV-3 cells were plated on fluorescently labeled gelatin (Alexa 568) before staining against TKS5 (green) and StarD13 (blue) and imaging using the 63x lens of the Zeiss fluorescent microscope. The merged magnified frames are pointed out on the original images as white boxes. Lower panels: SKOV-3 cells were plated on fluorescently labeled gelatin (Alexa 568) before staining against TKS4 (green) and StarD13 (blue) and imaging using the 63x lens of the Zeiss fluorescent microscope. The merged magnified frames are pointed out on the original images as white boxes. Scale bar is 10 μm. E. Left panel: Fixed selection line scan measurements along invadopodia of TKS4, StarD13, and Alexa 568 intensities from the perimeter to the center of the invadopodia. Right panel: Quantification of measurement of fluorescence intensities of TKS4, StarD13, and Alexa568 normalized to the lowest signal value across the ROI from the perimeter to the center of the invadopodia. F. Representative micrographs illustrating TKS5 expression (orange) and the ratiometric activation of Cdc42 FRET biosensor in SKOV3 cells. Higher magnifications of invadopodia illustrating TKS5 expression and the ratiometric activation of Cdc42 are shown on the right. Scale bar is 10 μm. G. Representative micrographs illustrating TKS4 expression (orange) and the ratiometric activation of Cdc42 FRET biosensor in SKOV3 cells. Scale bar is 10 μm. H. Representative micrographs illustrating StarD13 expression (orange) and the ratiometric activation of Cdc42 FRET biosensor in SKOV3 cells. Higher magnifications of invadopodia illustrating StarD13 expression and the ratiometric activation of Cdc42 are shown on the right. Scale bar is 10 μm. I. Left panel: Line scan measurements along invadopodia of StarD13 and FRET intensities from the perimeter to the center of the invadopodia. Right panel: Quantification of measurements of fluorescence intensities of StarD13 and FRET normalized to the lowest signal value across the ROI from the perimeter to the center of the invadopodia. J. Left panel: SKOV-3 cells were transfected with Luciferase siRNA. StarD13 siRNA or Cdc42 siRNA, immunostained against TKS5 (green) and TKS4 (magenta) and imaged using the 63x lens of the Zeiss fluorescent microscope. The scale bar is 10 μm. Right panel: Quantification of TKS5-positive and TKS4-positive invadopodia, respectively. The data are representative of the mean ± SEM of three independent experiments. Significance was set at p < 0.05.

**Fig. 9.**
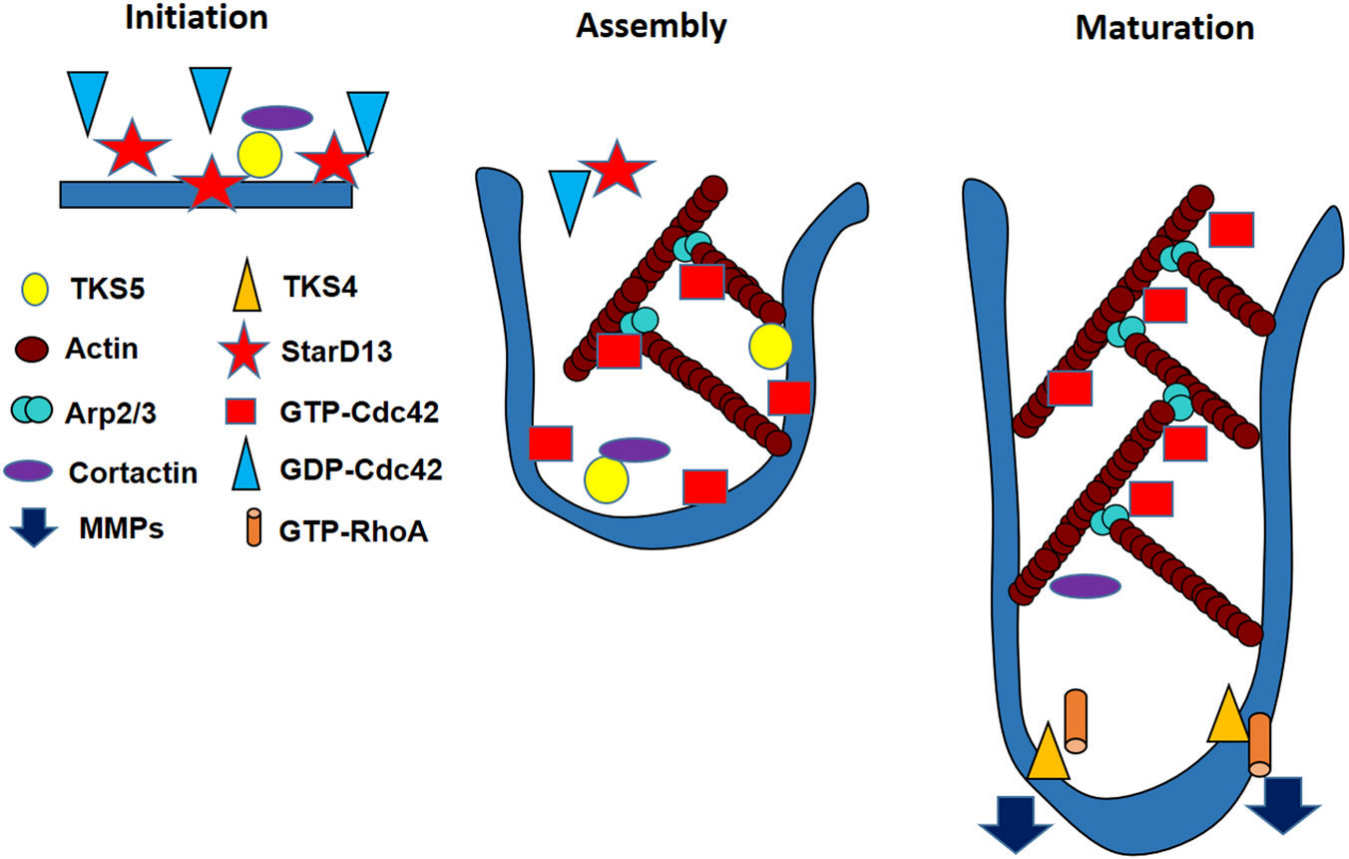
Model. During the initiation of invadopodia, RhoC and ROCK inhibit cofilin in the surrounding area (Bravo-Cordero et al., 2013) to keep the cofilin severing activity concentrated in the invadopodia area. Cofilin severs pre-existing actin filaments leading to the creation of freshly cut barbed ends and to initiate invadopodia. TKS5 helps aggregating proteins and as the invadopodia starts assembling, StarD13 evacuates for Cdc42 to activate. Cdc42 then activates N-WASP, which in turn activates Arp2/3, leading to actin branching and polymerization. TKS4, along with RhoA, recruits MMPs, which create mature invadopodia with matrix degradation ability.

Having isolated a subset of invadopodia which lacked the accumulation of GTP-Cdc42 (Fig. 7E), we sought to further characterize these invadopodia structures by immunostaining SKOV-3 for different markers that make different stages of invadopodia, mainly TKS5 and TKS4. TKS5 and TKS4 are both required for invadopodia formation and the invasive property for multiple human cancer cell lines, with TKS5 playing an early role in the assembly of invadopodia and TKS4 leading to the activation ECM degradation in mature invadopodia (Buschman et al., 2009; Courtneidge et al., 2005; Murphy and Courtneidge, 2011; Saykali and El-Sibai, 2014; Seals et al., 2005). Fig. 8A and B reveal two different populations of cortactin-labeled invadopodia in SKOV-3 cells: Cortactin/TKS5 double stained and Cortactin/TKS4 double stained invadopodia. Co-staining the cells with both TKS4 and TKS5 further proves that these two populations are, for the most part, distinct (Fig. 8C), whereby most invadopodia have either TKS4 or TKS5 staining and few have both (Supplemental Fig. S5). This could indicate the invadopodia in two stages of formation, where TKS5 localizes to the early stage and TKS4 to the late maturation stage (Fig. 9). In addition, the results indicate that some TKS5 positive invadopodia co-localize with StarD13 (Fig. 8D) and lack matrix degradation ability, but none of the TKS4 positive invadopodia (mature/with matrix degradation ability) express StarD13. This might indicate that, at a later stage of invadopodia formation (TKS4-positive invadopodia) StarD13 has to leave the site of invadopodia, in order for Cdc42 to activate (Fig. 8D and 8E). Interestingly, TKS5 invadopodia positive for StarD13 lack Cdc42 FRET activation whereas those which are negative for StarD13 exhibit Cdc42 FRET signal (Fig. 8F). Similarly, TKS4-positive invadopodia negative for StarD13 also display Cdc42 FRET signal (Fig. 8G). Fig. 8H and 8I also highlight that StarD13 never coincides with potentially active Cdc42 in invadopodia, thus suggesting an inhibitory role of StarD13 on Cdc42 in invadopodia.

Finally, we show that StarD13 depletion has no effect on the number of TKS5 positive invadopodia but that it significantly increases the number of TKS4 positive invadopodia as compared to the control (Fig. 8J). The opposite is observed following Cdc42 depletion, specifically, Cdc42 depletion has no effect on the number of TKS5 positive invadopodia but it significantly decreases the number of TKS4 positive invadopodia as compared to the control (Fig. 8J). This suggests a role of Cdc42 in the maturation of invadopodia and suggests that StarD13 suppresses the formation of mature invadopodia through the inhibition of Cdc42.

## 4. Discussion

This study provides an in-depth understanding of the role of StarD13 in ovarian cancer cells, as well as uncovers the key targets mediating StarD13 effects on cell proliferation, motility, invadopodia formation and invasion. Most, importantly, the data provide a model for the recruitment of an ordered set of molecules and signaling events required for the formation and the maturation of invadopodia in ovarian cancer cells. This suggests StarD13 as a main regulator of invadopodia and reveals, for the first time, its clear role in the spatial regulation of Cdc42 activation. Through the use of different markers of invadopodia, this study distinguishes stages of formation of invadopodia and the potential role of StarD13 and Cdc42 during these stages.

Immunohistochemistry and data microarray analysis revealed a lower expression of StarD13 in ovarian cancer tissues as compared to the normal ovarian tissues, which is similar to the results observed in lung and hepatocellular cancers (Al Haddad et al., 2020; Ching et al., 2003). StarD13 inhibition of cell proliferation is also in line with a potential tumor suppressor function of this protein (Al Haddad et al., 2020; El-Sitt et al., 2012; Hanna et al., 2014a; Khalil et al., 2014; Nasrallah et al., 2014; Yang et al., 2019b, 2019a; Zhang et al., 2017).

However, unlike in previous studies performed on breast, lung, astrocytoma and colorectal cancers (Al Haddad et al., 2020; Basak et al., 2018; El-Sitt et al., 2012; Hanna et al., 2014a; Khalil et al., 2014; Nasrallah et al., 2014), where StarD13 was found to be necessary for cell migration, it had no effect on the 2D motility in ovarian cancer cells. This is also in contradiction to reports where StarD13 was found to negatively regulate cell migration (Fan et al., 2012; Holeiter et al., 2008; Leung et al., 2005; Lin et al., 2010; Yang et al., 2019b). This could be due to the lack of effect on RhoA activity in ovarian cancer cells depleted of StarD13. In contrast, StarD13 depletion led to a notable and significant increase in GTP-RhoA in other tumors studied (Al Haddad et al., 2020; Ching et al., 2003; El-Sitt and El-Sibai, 2013; Jaafar et al., 2020; Khalil et al., 2014). This could be accounted for by a potential higher expression of Deleted in liver cancer-1 (DLC1), the StarD13 homolog, in ovarian cancer. DLC1 is a GAP for RhoA, which is often deleted in hepatocellular carcinoma and inactive in various types of human cancers including colon cancer (Liu et al., 2017). DLC1 is not deleted in ovarian carcinoma tissues, albeit expressed at a lower level than normal ovarian tissues (Ren et al., 2013), which could compensate for the GAP activity of StarD13. This could indicate that, while in other tumor types StarD13 is the main GAP for RhoA due to the absence of DLC1, in ovarian tumors DLC1 mainly drives this inhibition.

Knock down of StarD13 however, showed an increase in GTP-Cdc42, strongly suggesting that StarD13 is a GAP for this Rho GTPase in ovarian cancer cells, similarly to findings in other tumor types (El-Sitt et al., 2012; Hanna et al., 2014a; Khalil et al., 2014; Nasrallah et al., 2014). In addition, a slight increase in the adhesive ability of ovarian cancer cells was observed after StarD13 depletion. This is in agreement with previously reported decrease in adhesion/cell rounding in HepG2 hepatoma cells overexpressing DLC2 (Leung et al., 2005). Another main regulator of adhesion in cells is the Rho GTPase, Rac1. The depletion of StarD13 led to an increase in GTP-Rac1, which was due to a potential lack of Cdc42 inhibition (through the GAP activity). This is consistent with previous reports placing Cdc42 upstream from Rac1 in other cell types (El Atat et al., 2019; Yip et al., 2007). This also accounts for the increase in adhesion in StarD13-depleted cells, independently of RhoA, since knocking down Rac1 in StarD13-depleted cells reversed its effect on adhesion. While in other tumor types, an increase in adhesion and the disruption of adhesion dynamics inhibited cell motility (Al Haddad et al., 2020; Ching et al., 2003; El-Sitt and El-Sibai, 2013; Jaafar et al., 2020; Khalil et al., 2014), it did not affect the migratory ability of ovarian cancer cells. This discrepancy could be due to the distinct role adhesion turnover might play in migration in different cell types. Rac1 mainly drives the formation of point contacts. These are small punctate structures, found behind the front of the lamellipodium (Kaverina et al., 2002) and at the edge of cells. On their own, point contacts do not confer enough contractility the cells to move (Kaverina et al., 2002). Point contacts however, are precursors of focal adhesions, which are mediated by RhoA and provide the necessary mechanical strength for the cell bodies to contract and move forward (Gupton and Waterman-Storer, 2006). In these cells, the depletion of StarD13 leads to an increase in Rac1 activation and potentially an increase in the number of point contacts. Since RhoA activation is unchanged, however, the point contacts would remain in excess and, while would appear higher in number, would still be bottlenecked by the maturation rate-limiting step. Consequently, the detected increase in the number of adhesion structures is mostly reflecting structures that would not paralyze the cells through over-attachment to the matrix and would not decrease cell migration, as observed.

StarD13-depleted cells also showed a dramatic increase in ruffles and in protrusion (in quiesced cells as well as cells stimulated with EGF), which is mediated by Cdc42. This is consistent with previous reports highlighting the role of Cdc42 in actin polymerization and cell protrusion in cancer cells (El-Sibai et al., 2007). As mentioned above however, this did not affect the directionality of the cell undergoing migration. This could be due to additional spatial suppressive factors that concentrate the protrusion at the leading edge in migrating cells (Vega et al., 2011), whereas cells undergoing bath stimulation lose their directionality and a defined leading edge. Interestingly, in cells where Cdc42 was depleted, we observed concentrated ring-like structures rich in Arp2 and actin. This could be indicative of a lack of proper distribution of the Arp2/3 protein, due to the lack of activation of its organizer N-WASP (Rohatgi et al., 1999). RhoA was also required for matrix degradation and invasion, independently of StarD13.

In addition to the effect on actin-rich protrusion, StarD13 silencing increased matrix degradation and ovarian cancer cell invasion. This was previously observed in other tumor types (Al Haddad et al., 2020; Hanna et al., 2014a). More importantly, this was mediated by Cdc42. Indeed, silencing of Cdc42 reversed the increase observed in the StarD13-depleted cells. In addition, Cdc42 depletion completed abolished matrix degradation in control ovarian cancer cells. This is in line with the role of Cdc42 in promoting the production of metalloproteinases that degrade the extracellular matrix components facilitating the process of invasion (Stengel and Zheng, 2011; Yamaguchi et al., 2005). In a recent study, we showed that StarD13 depletion increases invadopodia formation through Cdc42 in normal lung cells and in lung cancer cells (Al Haddad et al., 2020). That corroborated the potential tumor suppressive function of StarD13 since, once depleted, normal lung cells recapitulated the tumor phenotype and formed invasive structures. In ovarian cancer cells, StarD13 depletion also led to an increase in cortactin-labeled and WASP-labeled potential invadopodia, while Cdc42 depletion completed abolished any of these structures. Cdc42 co-localized with the specific invadopodia marker TKS4. Cdc42 activation, observed through the Cdc42 FRET biosensor, also localized with TKS4 and TKS5 staining. Live imaging of Cdc42 activation through FRET, showed that StarD13 depletion leads to a higher and a more prolonged activation of Cdc42 in dots (potential invadopodia). This suggests a spatial (in invadopodia) and a temporal inhibition of Cdc42 by StarD13. This is reminiscent of the role of RhoC in the spatial regulation of cofilin in invadopodia (Bravo-Cordero et al., 2011). However, while RhoC depletion did not affect the number of invadopodia but inhibited their effectiveness, StarD13 depletion increased the number of invadopodia. In addition, the increase in the number of invadopodia in ovarian cancer cells could also potentially account for the slight increase in adhesion described earlier, because these structures are in essence podosome-type adhesion clusters (Block et al., 2008).

Interestingly, monitoring the dynamics of StarD13, Cdc42, TKS4 and TKS5 expression in cells and in invadopodia uncovered a set of organized events required for the formation and maturation of invadopodia (Fig. 9). The model proposed suggests that Cdc42 is recruited early to invadopodia but that it is not activated until StarD13 is evacuated. Cdc42 plays a direct role in the early recruitment of cortactin and WASP to the point of actin polymerization in invadopodia (as evidenced by the Cdc42 knock down in Fig. 7B). It is worth noting that RhoA activation in invadopodia is independent of StarD13 expression. Potentially, RhoA is activated in invadopodia to recruit MMPs and lead to matrix degradation as previously demonstrated (Chiang et al., 2016; Hoshino et al., 2009; Varon et al., 2006; Yu et al., 2013).

In an earlier study, we reported StarD13 to always be absent in invadopodial structures (Al Haddad et al., 2020). In that study, however, invadopodial structures were identified with TKS4 staining. In this study, we were able to identify two pools of invadopodia as labeled by TKS5 and TKS4, which co-localize with cortactin and mark early and late invadopodia, respectively (Fig. 9), as previously established in the literature (Murphy and Courtneidge, 2011). We also identified some TKS4/5 double-labeled invadopodia, which might be transitioning into maturation (Supplemental Fig. S5). StarD13 seemed to coincide with TKS5 labeling in some of these structures, which is indicative of early invadopodia where StarD13 has not yet evacuated. Indeed, Fig. 8F shows that, while a subset of the TKS5-labeled invadopodia show Cdc42 activation through the FRET signal, others do not. The TKS5 positive invadopodia where Cdc42 has not activated yet must be short lived since, as mentioned earlier, Cdc42 plays an early role in the recruitment of cortactin and WASP (Fig. 7B). Indeed, the kinetics of Cdc42 activation to invadopodia-like structures in Fig. 7E showed a very quick activation of Cdc42 (reflected by the high FRET ratio) following its localization (reflected by the low FRET ratio) through the duration of the invadopodia lifetime.

In summary, both RhoA and Cdc42 activation persist in invadopodia while StarD13 is only expressed in early invadopodia along with TKS5 and never coincides with TKS4 (Fig. 8D and E). Interestingly, when StarD13 is knocked down we observe the same number of TKS5 positive invadopodia but more TKS4 positive invadopodia, which is due to the increase in GTP-Cdc42 and which coincides with the increase in matrix degradation. In contrast, Cdc42 knock down abrogates the formation of TKS4 positive invadopodia. Collectively, this suggests that Cdc42 might be required for the recruitment of TKS4 to invadopodia to mature. Coincidentally, TKS4 has been previously found to be necessary for the recruitment of MT1-MMP to invadopodia (Murphy and Courtneidge, 2011). At the same time, this was found to be regulated by Cdc42 (Noritake et al., 2005; Sakurai-Yageta et al., 2008). Here we suspect a novel involvement of Cdc42 in matrix degradation in invadopodia through the recruitment of TKS4, in addition to its early role in WASP activation and actin polymerization.

Altogether this study showed that StarD13 is a GAP for Cdc42 and emphasized the importance of StarD13 and Cdc42 in regulating the formation of invadopodia in a temporal and functional manner.

## Supplementary Material

Supplemental Movie S5

Supplemental Movie S7

Supplemental Movie S6

Supplemental Movie S8

Supplemental Movie S3

Supplemental Movie S2

Supplemental Movie S1

Supplemental Movie S4

Supplemental Figure S5

Supplemental Figure S1

Supplemental Figure S4

Supplemental Figure S2

Supplemental Figure S3

## Figures and Tables

**Table 1:** StarD13 depletion does not affect the motility of ovarian cancer cells.

	SKOV-3		Caov-3	
	Total path (μm)	Speed (μm/ min)	Total path (μm)	Speed (μm/ min)
Luciferase siRNA	48.2	0.401666667	35.7	0.2975
StarD13 siRNA (Oligo1)	46.98	0.3915	36.4	0.303333333
