# Supplementary figures and images for: StarD13 negatively regulates invadopodia formation and invasion in high-grade serous (HGS) ovarian adenocarcinoma cells by inhibiting Cdc42
Source: Eur J Cell Biol. Author manuscript; Available in PMC 2023 Jan 1. (PMC8756770; doi:10.1016/j.ejcb.2021.151197)

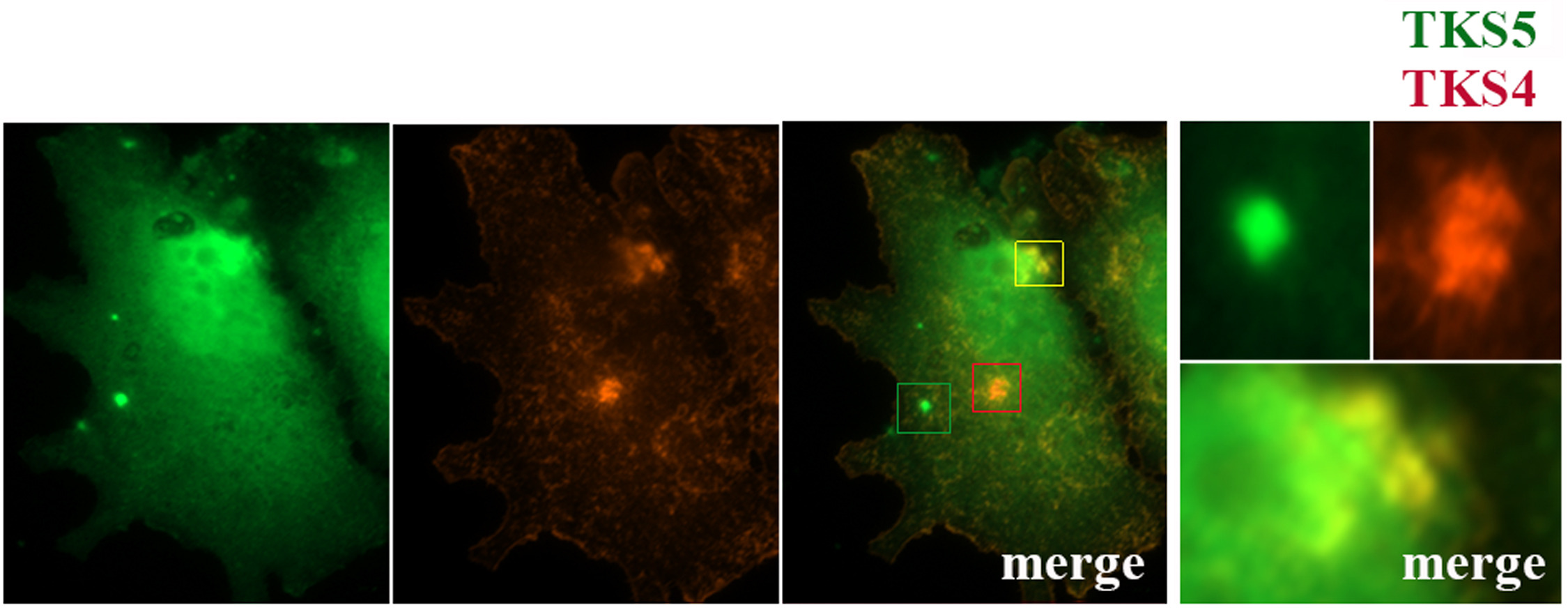

Supplement: Supplemental Figure S5 [file NIHMS1768151-supplement-Supplemental_Figure_S5.jpg]

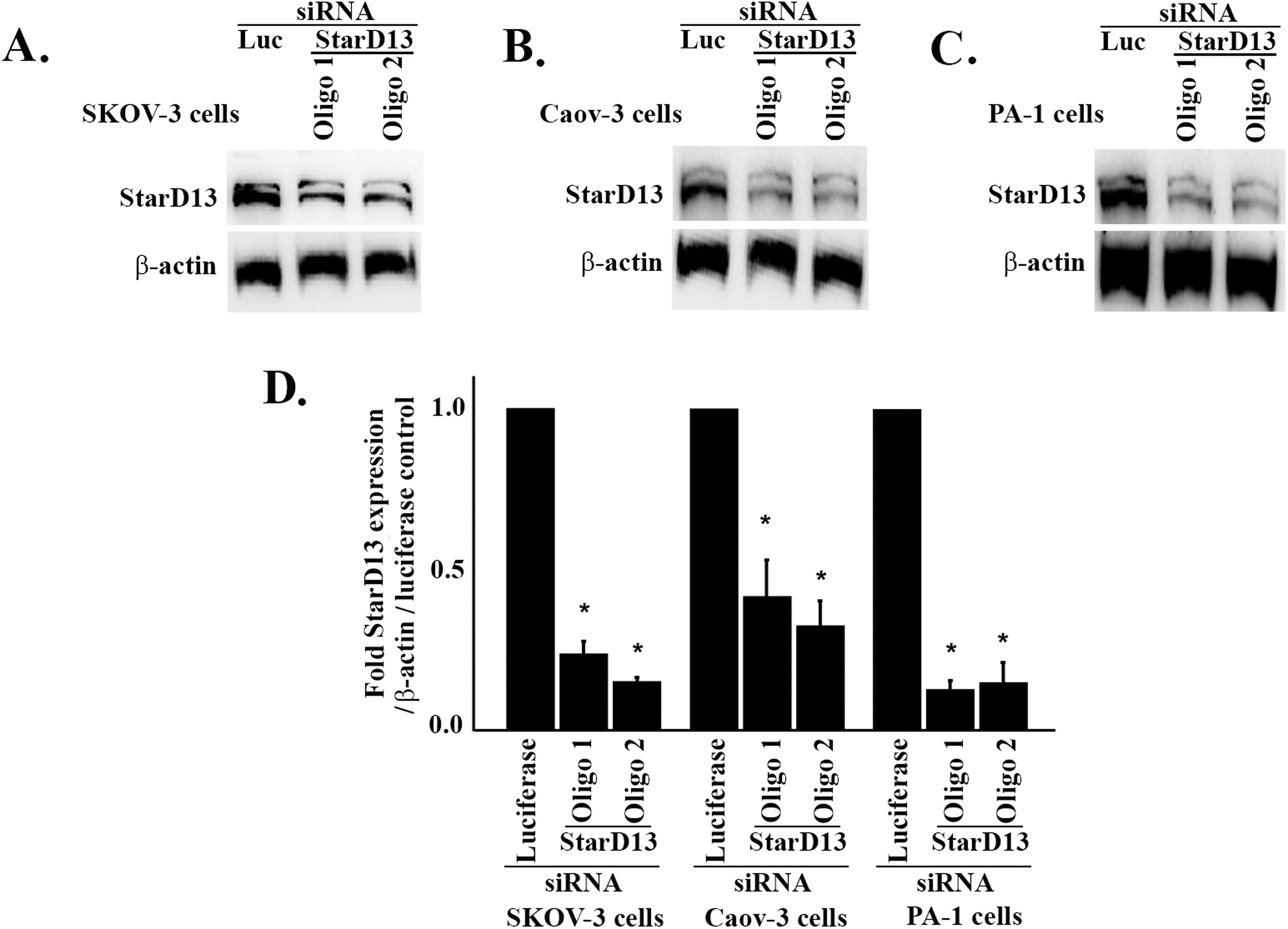

Supplement: Supplemental Figure S1 [file NIHMS1768151-supplement-Supplemental_Figure_S1.jpg]

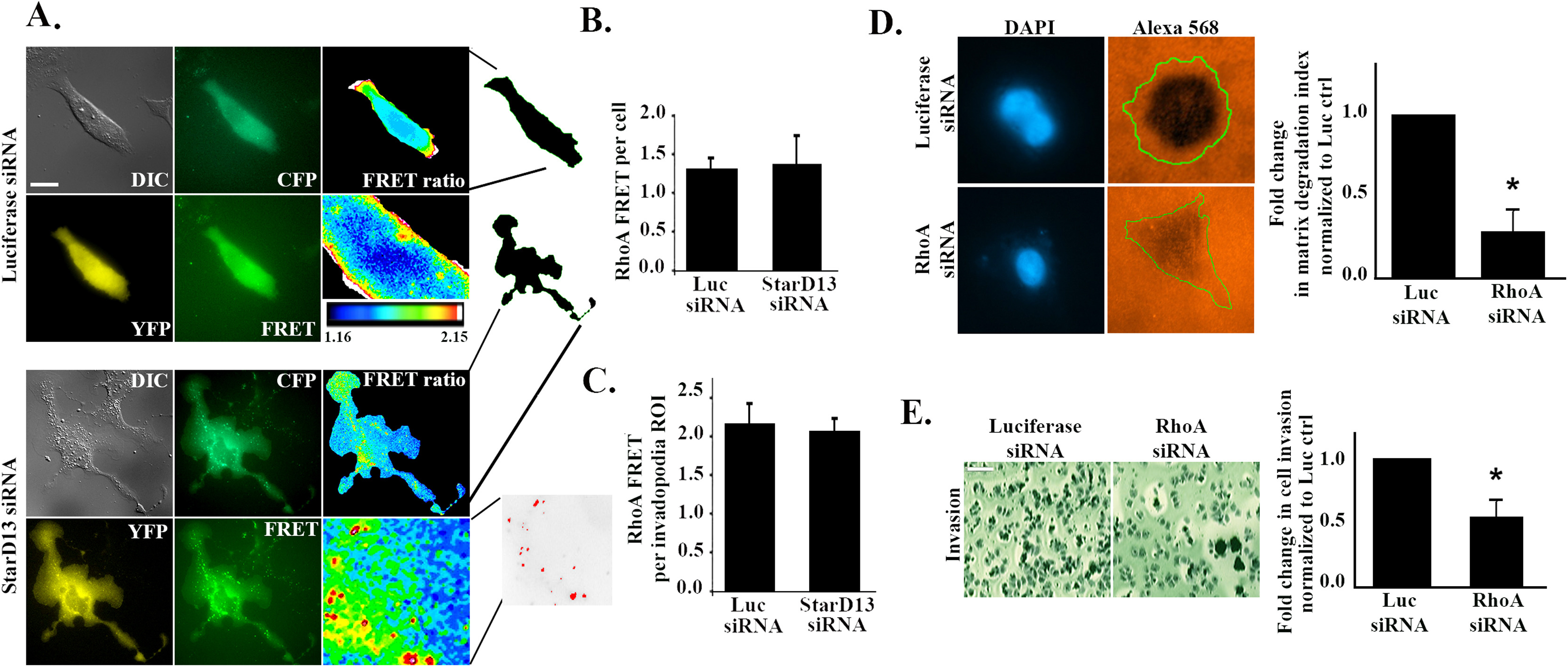

Supplement: Supplemental Figure S4 [file NIHMS1768151-supplement-Supplemental_Figure_S4.jpg]

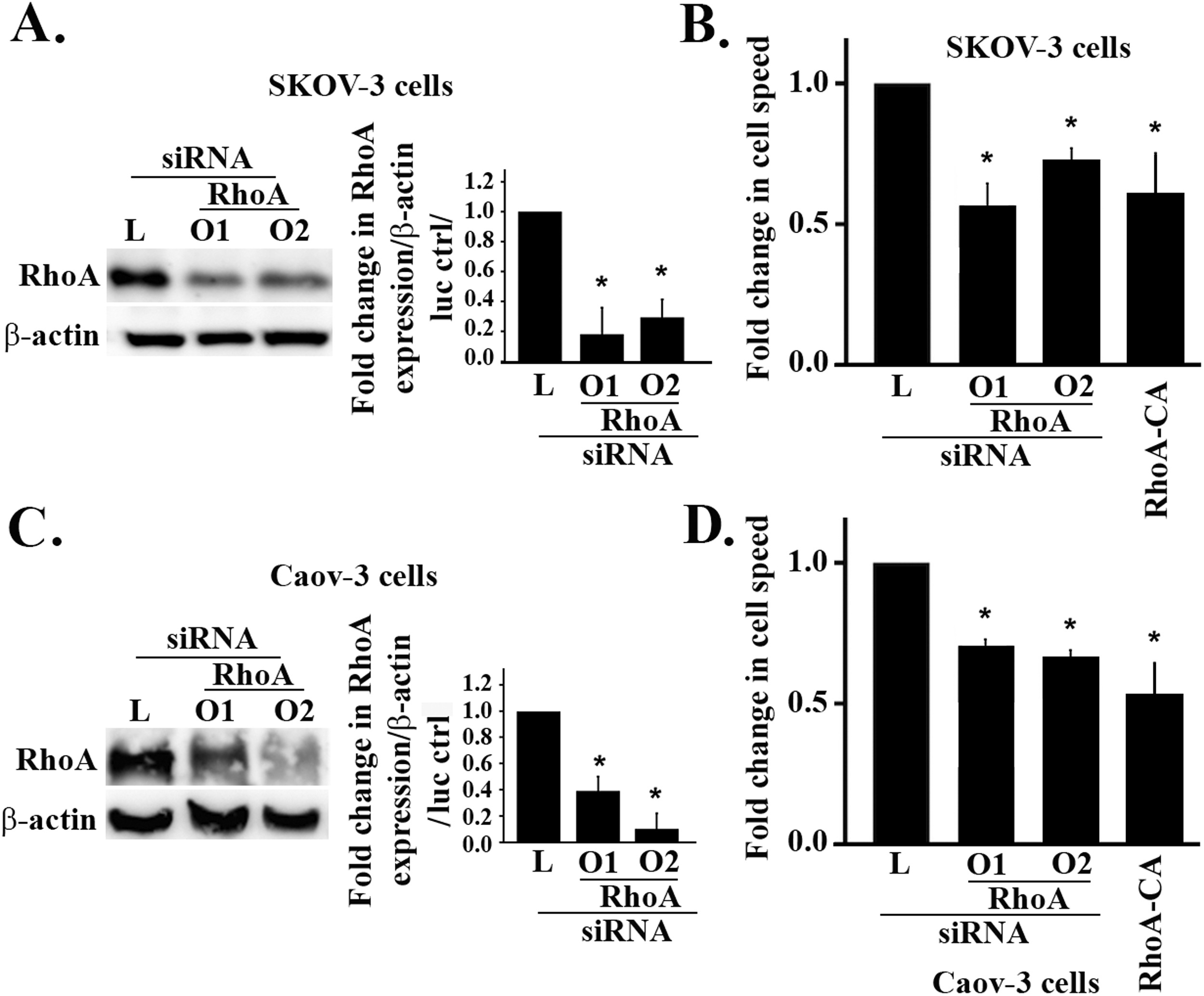

Supplement: Supplemental Figure S2 [file NIHMS1768151-supplement-Supplemental_Figure_S2.jpg]

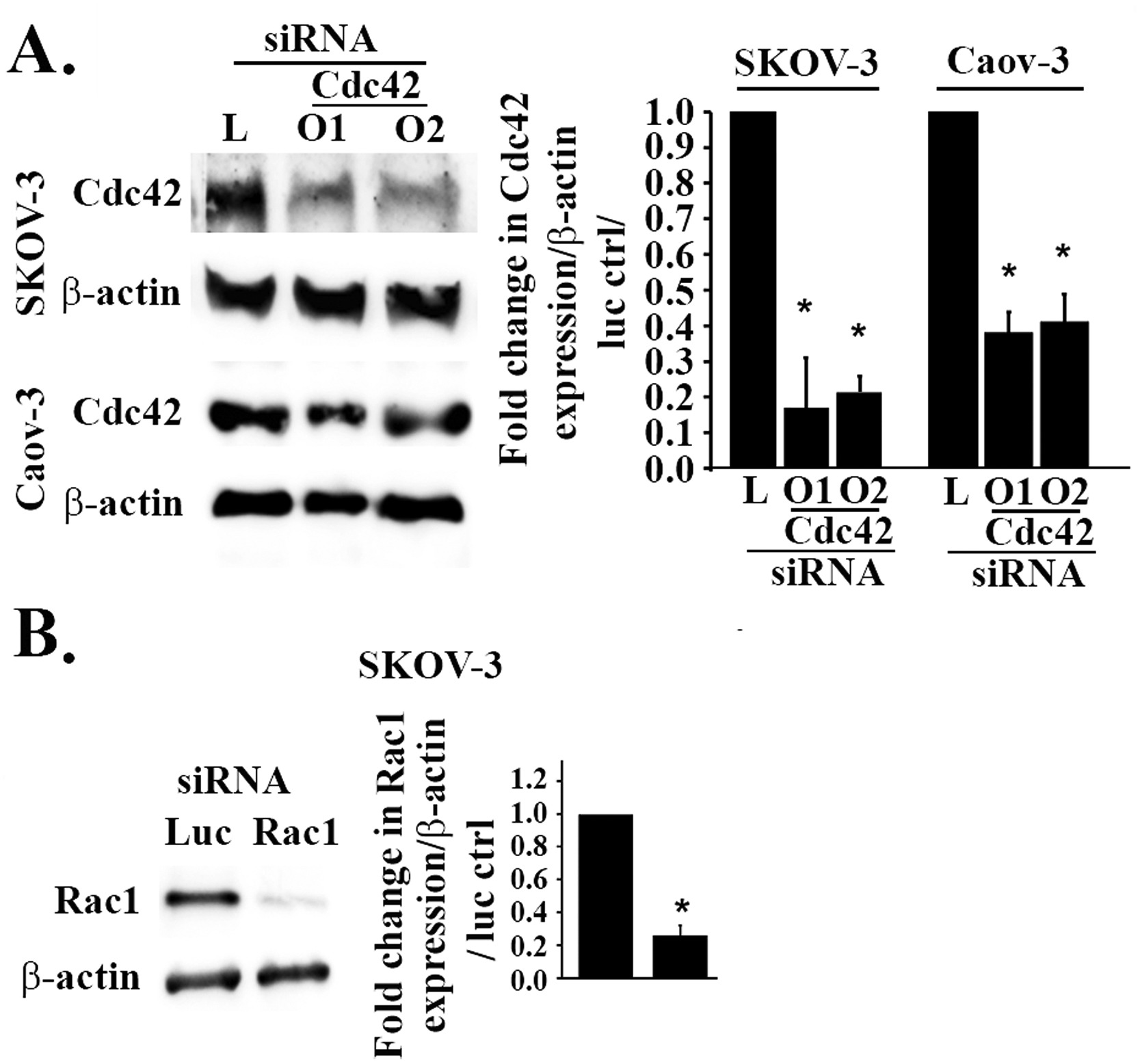

Supplement: Supplemental Figure S3 [file NIHMS1768151-supplement-Supplemental_Figure_S3.jpg]
